# MFP-MFL: Leveraging Graph Attention and Multi-Feature Integration for Superior Multifunctional Bioactive Peptide Prediction

**DOI:** 10.3390/ijms26031317

**Published:** 2025-02-04

**Authors:** Fang Ge, Jianren Zhou, Ming Zhang, Dong-Jun Yu

**Affiliations:** 1 State Key Laboratory of Flexible Electronics (LoFE), Institute of Advanced Materials (IAM), Nanjing University of Posts and Telecommunications, 9 Wenyuan Road, Nanjing 210023, China; gfang0616@njupt.edu.cn; 2 School of Computer, Jiangsu University of Science and Technology, 666 Changhui Road, Zhenjiang 212100, China; 221210701330@stu.just.edu.cn (J.Z.); zhangming@just.edu.cn (M.Z.); 3 School of Computer Science and Engineering, Nanjing University of Science and Technology, 200 Xiaolingwei, Nanjing 210094, China

**Keywords:** bioactive peptides, multifunctional peptides, graph attention network, protein language model, ensemble method

## Abstract

Bioactive peptides, composed of amino acid chains, are fundamental to a wide range of biological functions. Their inherent multifunctionality, however, complicates accurate classification and prediction. To address these challenges, we present MFP-MFL, an advanced multi-feature, multi-label learning framework that integrates Graph Attention Networks (GAT) with leading protein language models, including ESM-2, ProtT5, and RoBERTa. By employing an ensemble learning strategy, MFP-MFL effectively utilizes deep sequence features and complex functional dependencies, ensuring highly accurate and robust predictions of multifunctional peptides. Comparative experiments demonstrate that MFP-MFL achieves precision, coverage, and accuracy scores of 0.799, 0.821, and 0.786, respectively. Additionally, it attains an Absolute true of 0.737 while maintaining a low Absolute false of 0.086. A comprehensive case study involving 86,970 mutations further highlights the model’s ability to predict functional changes resulting from sequence variations. These results establish MFP-MFL as a powerful tool for the discovery and application of multifunctional peptides, offering significant potential to advance research and biomedical applications.

## 1. Introduction

Bioactive peptides, synthesized by covalently linking natural or synthetic amino acids via peptide bonds, display distinct biological activities and execute a range of physiological functions. Consequently, their investigation has emerged as a cutting-edge area in contemporary life sciences [1]. These peptides are distinguished by their structural complexity and diversity, and they are ubiquitously present in animals, plants, microorganisms, and synthetically engineered organisms. They exhibit a multitude of biological functions, including antimicrobial, antidiabetic, antihypertensive, anti-inflammatory, antiviral, antitumor, antioxidant, and immunomodulatory activities, rendering them highly valuable for applications in medicine, agriculture, and the food industry [2,3]. Nonetheless, the precise and efficient determination of bioactive peptide functions remains a considerable challenge due to their functional intricacy and diversity.

Traditional experimental methodologies have been pivotal in elucidating the functions of bioactive peptides. However, their inherent limitations—namely, time consumption, labor intensiveness, and high costs—render them inadequate to satisfy the demands of modern research [4]. To address these limitations, computational approaches have made significant strides in predicting bioactive peptide functions, thereby markedly enhancing research efficiency and predictive accuracy [5].

Computational methodologies for bioactive peptide analysis are primarily categorized by their specific objectives. Initially, sequence analysis and feature extraction focus on the close relationship between peptide functionality and their amino acid sequences. This analysis facilitates the identification of crucial sequence motifs linked to particular functions, with the integration of machine learning and deep learning techniques significantly advancing this field [6]. Techniques such as support vector machines (SVM), random forests (RF), and convolutional neural networks (CNN) are extensively employed to extract and evaluate peptide sequence features for predicting biological activities [7]. Recently, transformer-based deep learning models have made significant breakthroughs in protein sequence analysis, including BERT [8], RoBERTa [9], and ESM-2 [10], which have achieved notable progress in protein sequence analysis [11]. These models adeptly capture intricate sequence patterns and features from extensive peptide datasets, enabling precise function prediction and markedly reducing the need for experimental validation.

Secondly, the three-dimensional conformation of peptides is pivotal in determining their biological functions. Advances in deep learning, particularly with models like AlphaFold 2 [12], have reached unprecedented accuracy in predicting protein and peptide structures. These high-resolution structural predictions offer deeper insights into the functional mechanisms of bioactive peptides and support experimental validation efforts. Additionally, data-driven strategies that integrate diverse biological data sources—encompassing sequence, structural, and functional information—have emerged as robust tools. Ensemble learning models that combine sequence and structural features further enhance prediction accuracy and robustness [13,14].

Despite these advancements, predicting the functions of multifunctional peptides remains challenging due to their inherent complexity, diversity, and the limitations of current computational frameworks [15]. The functionality of multifunctional peptides is determined by both their amino acid sequences and three-dimensional structures. While existing models efficiently process large-scale sequence data, they often fail to account for the diversity and functional overlap characteristic of multifunctional peptides [16]. Additionally, most machine learning and deep learning models are tailored for single-function predictions, complicating the accurate identification and differentiation of multiple functionalities. Moreover, challenges such as data scarcity and quality issues further complicate the research landscape. Predictive models for bioactive peptides rely on high-quality experimental data [17], yet available datasets frequently suffer from imbalance and noise. Variations in experimental conditions and techniques exacerbate inconsistencies, undermining data comparability and model performance.

Pre-trained protein language models (pLMs) present a promising solution to these challenges. By pre-training on millions or even tens of millions of protein sequences, these models effectively capture the intricate relationships between sequences and their biological properties, thereby significantly enhancing both the accuracy and efficiency of multifunctional peptide predictions [18].

In this study, we introduce MFP-MFL, an innovative multi-feature, multi-label learning framework designed to address the critical challenges in predicting multifunctional peptides. MFP-MFL reformulates the multi-label prediction problem as a graph node classification task, leveraging multidimensional features of peptide sequences and employing Graph Attention Networks (GAT) for efficient modeling. Initially, pre-trained protein language models such as ESM-2 [10], ProtT5 [19], and RoBERTa [9] are utilized to extract deep peptide sequence features, ensuring the representation of rich biological information across diverse feature spaces. Subsequently, an ensemble learning strategy integrates predictions from multiple models to enhance accuracy and generalizability. Compared to traditional single-feature learning approaches, MFP-MFL effectively captures the intricate structural and functional relationships within peptide sequences, demonstrating significant advantages in predicting multifunctional peptides. Experimental results confirm that MFP-MFL achieves high predictive accuracy while maintaining robustness and stability, validating its practical value and potential as a computational tool for peptide function analysis.

Therefore, the key contributions of this work are as follows: (1) This study combines three advanced protein language models (including ESM-2, ProtT5, and RoBERTa) to efficiently extract deep functional features from peptide sequences. This approach overcomes the limitations of traditional methods in feature representation, offering a novel framework for advancing research on multifunctional bioactive peptides. (2) To capture the complex correlations between peptide functional labels, we introduce a Graph Attention Network (GAT) as the core model. By effectively modeling label interdependencies, this approach significantly enhances the model’s performance in multifunctional prediction tasks, presenting a new perspective on addressing multifunctional correlations. (3) Through extensive experimental evaluations, we demonstrate the significant advantages of MFP-MFL across several key metrics. In comparison with both traditional machine learning and deep learning methods, MFP-MFL consistently outperforms existing techniques in terms of prediction accuracy and stability. This highlights the clear technical superiority of the framework for tackling complex multifunctional bioactive peptide prediction tasks. (4) The flexibility of the MFP-MFL framework is further validated in predicting functional changes resulting from peptide sequence mutations, as evidenced by large-scale case studies.

## 2. Results and Discussions

This section delineates the experimental outcomes of various models and methodologies. Multiple feature sets were employed for training and prediction, and optimal model ensembles were determined through weighted adjustments in ensemble learning. The MFP-MFL model was evaluated against leading classifiers for multifunctional bioactive peptides. All experiments were conducted using PyTorch 2.1.1 and executed on an NVIDIA GeForce RTX 4090 GPU.

To mitigate label imbalance in bioactive peptide datasets, where certain labels have limited samples, multi-label instances were strategically grouped to achieve a balanced partitioning of the dataset. This approach enhanced the stability of model training and improved the reliability of performance evaluations. Detailed evaluation metrics are provided in Text S1.

### 2.1. Performance Comparative of ESM-2, ProtT5, and RoBERTa Embeddings

This study assesses the impact of different feature embeddings on the classification of multifunctional peptides by comparing ESM-2, ProtT5, and RoBERTa using the GAT model as a baseline model. To ensure robust and reliable results, each embedding was subjected to 10 independent training and testing iterations, with the outcomes summarized in Table S1.

Among the three embeddings, RoBERTa consistently demonstrated superior performance across all evaluation metrics. Specifically, it achieved a precision of 0.784 ± 0.003, coverage of 0.800 ± 0.009, accuracy of 0.770 ± 0.003, Absolute true of 0.727 ± 0.004, and Absolute false of 0.093 ± 0.001. These findings underscore the efficacy of RoBERTa embeddings in accurately capturing functional characteristics within peptide sequences.

**Figure 1:**
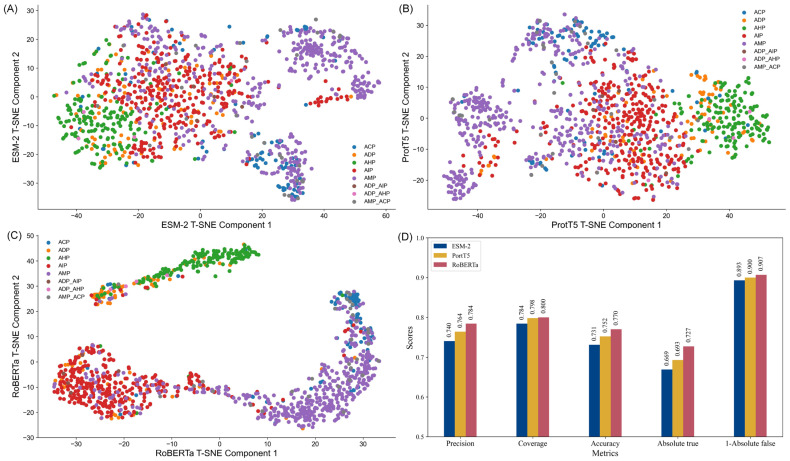
Comparative analysis of feature embeddings performance. (**A**–**C**) t-SNE dimensionality reduction visualizations for ESM-2, PortT5, and RoBERTa embeddings. (**D**) Evaluation metrics for ESM-2, PortT5, and RoBERTa embeddings. Abbreviation: anti-microbial peptide (AMP), anti-cancer peptide (ACP), anti-diabetic peptide (ADP), anti-hypertensive peptide (AHP), anti-inflammatory peptide (AIP).

To illustrate the comparative performance, Figure 1D depicts a bar graph of each embedding metric. These visualizations clearly highlight RoBERTa’s dominance across all metrics. Additionally, to explore the structural differences between the embeddings, t-SNE (t-distributed stochastic neighbor embedding) was employed to reduce the feature space’s dimensionality. This technique facilitates the projection of high-dimensional data into a two-dimensional space, enabling the intuitive visualization of data clusters and feature relationships.

The t-SNE plots (Figure 1A–C) reveal distinct clustering patterns for each embedding. RoBERTa exhibited the most well-defined clusters, particularly for categories such as anti-inflammatory peptides (AIP) and anti-microbial peptides (AMP), demonstrating its strong discriminative capability. In contrast, ESM-2 embeddings displayed moderate clustering, with some overlap between categories like anti-diabetic peptides (ADP) and anti-inflammatory peptide (AIP), indicating limited precision in distinguishing subtle functional differences. ProtT5 embeddings, while showing a more uniform distribution, lacked clear clustering patterns, suggesting an ability to capture global trends but with reduced category separation. These observations indicate that each embedding type possesses unique strengths, with RoBERTa providing the most robust discriminative performance, thereby offering valuable insights for the design of peptide classification models.

In addition, we used the traditional KNN method as a baseline to predict the performance of different feature embeddings, with the detailed results provided in Tables S2 and S3. Notably, RoBERTa features consistently outperformed both ESM-2 and ProtT5 features, achieving the highest prediction accuracy. These results further reinforce the effectiveness of RoBERTa features in peptide prediction tasks.

Subsequently, ensemble modeling was employed to integrate multiple embeddings, with the results detailed in Table 1. The multi-feature ensemble model achieved significant improvements over single-feature models, attaining a precision of 0.799, coverage of 0.821, and accuracy of 0.786. These results indicate that feature integration effectively enhances both classification precision and coverage. Moreover, the ensemble model achieved an Absolute true of 0.737 and Absolute false of 0.086, reflecting substantial improvements in correctly identifying positive samples and a notable reduction in misclassifying negative samples.

In conclusion, the results presented in Table 1 validate the effectiveness of the multi-feature fusion strategy. By integrating diverse embeddings, the GAT model achieved significant advancements in predictive performance across all metrics, providing a highly efficient and reliable framework for multifunctional peptide classification tasks. Additionally, to facilitate a more precise analysis of the prediction results, we computed the confusion matrices for each label (available in Table S4). This step further strengthens the reliability of the experimental findings.

### 2.2. Evaluation of GAT, MLP, and GCN with Multi-Feature Integration

**Figure 2:**
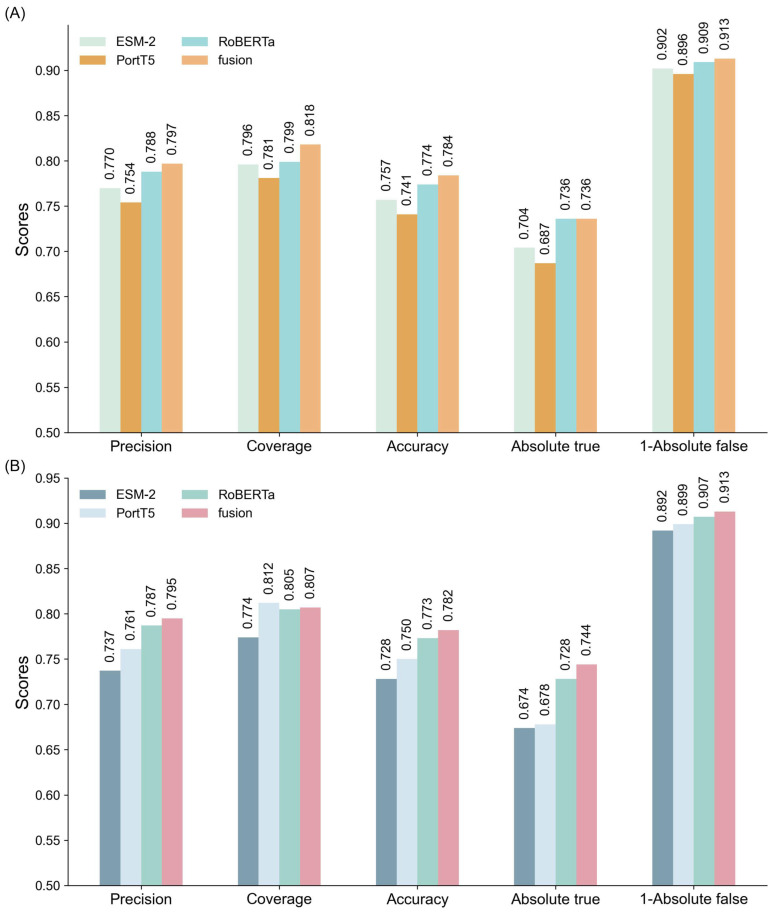
Comparative performance evaluation of feature extraction and ensemble learning on (**A**) MLP and (**B**) GCN Models.

To comprehensively evaluate the performance of different models in classifying multifunctional peptides, this section compares GAT networks, MLP, and GCN. The comparison is based on experimental results and theoretical considerations, focusing on each model’s ability to integrate single and multi-feature ensembles. All models were trained under identical parameter settings, with detailed results summarized in Figure 2 and Tables S5 and S6. As depicted in Figure 2, both MLP and GCN models exhibit significant performance enhancements when utilizing multi-feature ensembles. However, their performance remains inferior to that of the GAT model across all evaluation metrics.

For the MLP model (Figure 2A), the multi-feature ensemble achieved a precision of 0.797, coverage of 0.818, and accuracy of 0.784, indicating substantial improvements over single-feature models. The corresponding Absolute true and Absolute false were 0.736 and 0.087, respectively, demonstrating the model’s ability to reliably identify positive samples while effectively minimizing false positives. Despite these advancements, the MLP model faces challenges in handling complex feature interactions, resulting in a performance that is less effective compared to the GAT model.

In the case of the GCN model (Figure 2B), a similar trend is observed. The multi-feature ensemble attained a precision of 0.795, coverage of 0.807, and accuracy of 0.782, along with an Absolute true of 0.744 and Absolute false of 0.087. These results illustrate that while GCN benefits from multi-feature integration, the gains are limited compared to the GAT model, particularly in coverage and accuracy, where GCN shows marginally lower performance.

Conversely, the GAT model (Table 1) consistently outperformed both MLP and GCN models across all evaluation metrics. Its multi-feature ensemble achieved a precision of 0.799, coverage of 0.821, and accuracy of 0.786. Additionally, the model recorded an Absolute true of 0.737 and Absolute false of 0.086, highlighting its superior capability to accurately capture positive samples while minimizing false negatives. These results underscore GAT’s proficiency in leveraging complementary information across features, leading to enhanced classification accuracy and robustness.

The comparative evaluation underscores that GAT’s graph-based architecture enables it to model complex dependencies and complementary relationships between features more effectively than MLP and GCN. This capability significantly boosts the performance of multi-feature ensembles. Furthermore, GAT demonstrated exceptional robustness and stability, particularly in handling imbalanced datasets with unequal distributions of positive and negative samples.

Consequently, selecting GAT as the core model for multifunctional peptide classification is strongly supported by both experimental data and theoretical foundations. Additionally, feature fusion experiments were conducted across all models, with the results detailed in Supplementary Tables S7–S9. These experiments revealed that feature fusion did not yield significant performance gains, likely due to the fusion strategies’ inability to fully exploit the unique strengths of individual features. Furthermore, the integration process may have led to the loss of critical information, thereby explaining the limited improvements observed.

### 2.3. Comparison of MFP-MFL with Existing Prediction Methods

**Figure 3:**
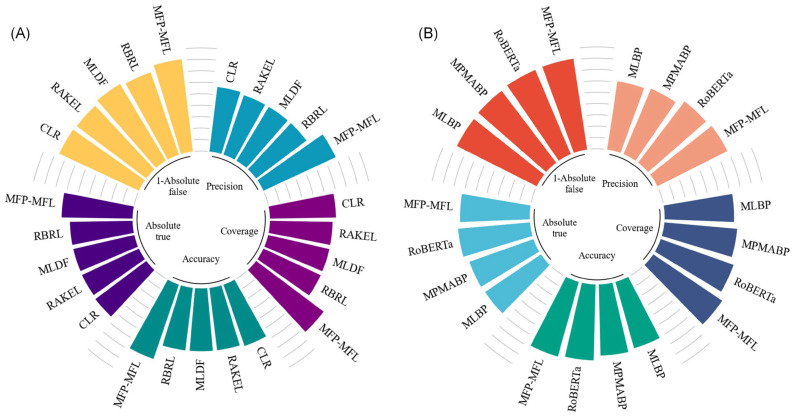
Comparative performance of (**A**) machine learning and (**B**) deep learning models in multifunctional peptide classification.

To rigorously assess the performance of the MFP-MFL approach, we compared it against a range of state-of-the-art prediction methods. These methods are categorized into two groups: machine learning-based approaches, including CLR [20], RAKEL [21], MLDF [22], and RBRL [23], and deep learning-based approaches, including MLBP [24], MPMABP [25], and RoBERTa [26]. MLBP [24] and MPMABP [25] utilize RNN and CNN architectures for classifying multi-functional peptides, while RoBERTa [26] employs a pre-trained large language model for feature extraction and classification. Although the iMFP-LG [27] model was initially considered, it was excluded from the final analysis due to identified issues with dataset partitioning. The details of the aforementioned model are available in Text S4. The detailed experimental results are presented in Table S10, while the key performance comparisons are illustrated in Figure 3.

As depicted in Figure 3A, MFP-MFL demonstrates clear superiority over machine learning-based methods. Specifically, MFP-MFL achieves precision, coverage, and accuracy scores of 0.799, 0.821, and 0.786, respectively, significantly outperforming CLR, RAKEL, MLDF, and RBRL. For instance, RBRL achieves corresponding scores of 0.650, 0.651, and 0.649, which are 14.9%, 17%, and 13.7% lower than those of MFP-MFL. Furthermore, MFP-MFL attains Absolute true and Absolute false of 0.737 and 0.086, respectively, surpassing other machine learning-based methods. Compared to RBRL, MFP-MFL improves the Absolute true by 9.1% and reduces the Absolute false by 5.4%. These results underscore the robust performance of MFP-MFL among ML-based approaches, particularly in its capacity to identify true positive samples and minimize false positives.

Figure 3B highlights the comparative performance of MFP-MFL against deep learning-based methods. MFP-MFL achieves precision, coverage, and accuracy scores of 0.799, 0.821, and 0.786, respectively, outperforming RoBERTa, which achieves 0.781, 0.786, and 0.769, reflecting improvements of 1.8%, 3.5%, and 1.7%. Although MFP-MFL’s Absolute true (0.737) is slightly lower than that of RoBERTa (0.741), its Absolute false (0.086) is superior to RoBERTa (0.092), indicating its enhanced capability to mitigate false positive classifications. These findings suggest that MFP-MFL excels in classification precision and coverage and also demonstrates exceptional robustness in handling imbalances between positive and negative samples.

Overall, the MFP-MFL model achieves superior prediction performance on the MFBP dataset compared to the existing computational methods, such as CLR and MLBP. These outcomes affirm the practical effectiveness and theoretical advantages of MFP-MFL in multifunctional peptide analysis, establishing it as a reliable and innovative solution for advancing research in this domain.

### 2.4. Evaluation of Multi-Label Methods on Single-Function Bioactive Peptides

**Figure 4:**
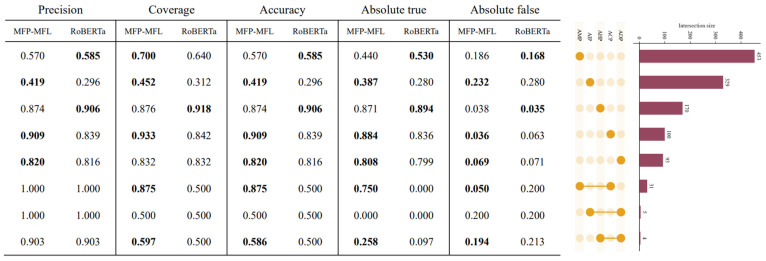
Comparative performance assessment of MFP-MFL and benchmark model on single-function and multifunctional bioactive peptide classification. The bold data indicate the optimal values for each indicator within a specific category. In the figure, the yellow shading denotes the specific category, while the red shading represents the number of instances within that category.

To comprehensively evaluate the performance of the MFP-MFL model, we assessed its predictive capabilities on both single-function and multi-function peptide datasets. The results consistently demonstrate that MFP-MFL surpasses existing state-of-the-art methods across most classification tasks, showcasing exceptional robustness and reliability in multi-function peptide classification. For instance, as illustrated in Figure 4, MFP-MFL achieved an accuracy of 0.875 in the ACP_AMP category, highlighting its proficiency in accurately identifying complex multifunctional peptides. In contrast, RoBERTa exhibited higher precision in specific single-function peptides, such as AHP and AMP, indicating its enhanced sensitivity to certain functional categories.

**Figure 5:**
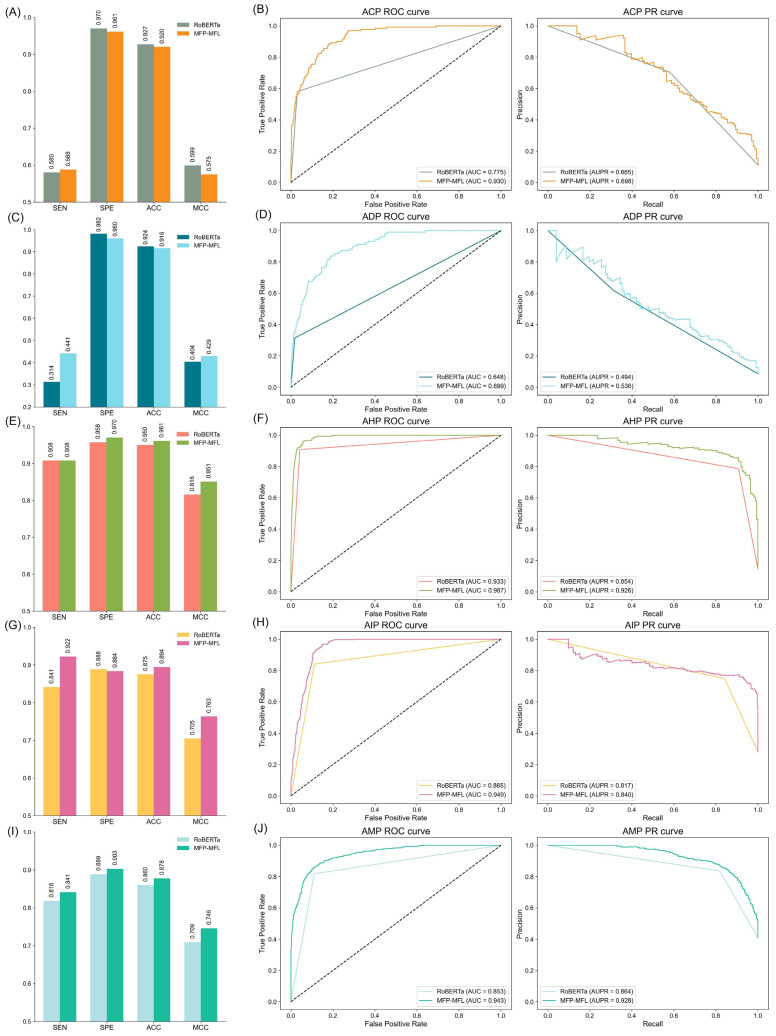
Comparative analysis of MFP-MFL and RoBERTa models: classification performance metrics and ROC/PR curve evaluations across ACP, ADP, AHP, AIP, and AMP sub-datasets. (**A**,**C**,**E**,**G**,**I**) illustrate the classification performance metrics. (**B**,**D**,**F**,**H**,**J**) present the ROC and PR curves for each respective sub-dataset, where the dashed line in each sub-figure represents the performance baseline of the random classifier. Note: the data presented in this figure are computed using the binary classification methodology outlined in Text S1.

In single-function peptide experiments, peptides exhibiting a specific function were classified as positive samples, while others were designated as negative. Subsequently, classification assessments were performed on five functional datasets: ACP, ADP, AHP, AIP, and AMP. As illustrated in Figure 5 and detailed in Tables S11 and S12 (Table S16 is the confusion matrix of the RoBERTa model), MFP-MFL consistently outperformed RoBERTa across key metrics, including SEN, specificity SPE, ACC, MCC, and AUC, demonstrating its superior predictive performance.

MFP-MFL showed a significant advantage in capturing functional peptide signals, evidenced by its consistently higher SEN, ACC, MCC, and AUC values. For instance, in the ACP dataset, MFP-MFL achieved an AUC of 0.930, markedly surpassing RoBERTa’s 0.775, along with a SEN of 0.588 and MCC of 0.575. Although RoBERTa exhibited slightly higher SPE (0.970) and ACC (0.927) compared to MFP-MFL’s 0.961 and 0.920, the balanced performance of MFP-MFL underscores its robustness. Similarly, in the AMP dataset, MFP-MFL attained an AUC of 0.943 versus RoBERTa’s 0.853, alongside improvements in SEN (0.841), SPE (0.903), and MCC (0.746), highlighting its reliability in identifying antimicrobial peptides.

While MFP-MFL excelled in most metrics, RoBERTa achieved comparable or slightly higher SPE in certain datasets, such as ADP (0.982) and AIP (0.888). However, MFP-MFL outperformed RoBERTa in the ADP dataset with an AUC of 0.899 compared to RoBERTa’s 0.648, along with higher SEN (0.441) and MCC (0.429). In the AIP dataset, MFP-MFL achieved a superior AUC of 0.949 versus RoBERTa’s 0.865, as well as increased SEN (0.922) and MCC (0.763). Furthermore, in the AHP dataset, MFP-MFL reached an AUC of 0.987, surpassing RoBERTa’s 0.933, with enhanced SPE (0.970) and MCC (0.851). These comparisons emphasize MFP-MFL’s robust capability in distinguishing functional peptides across diverse datasets.

In summary, the results are consistent and demonstrate that MFP-MFL surpasses RoBERTa in single-function peptide classification across all five datasets. Its consistent achievement of high AUC, sensitivity, accuracy, and MCC values reflects its robustness and versatility as a predictive model. Notably, substantial improvements in critical metrics such as AUC and MCC highlight MFP-MFL’s efficacy in accurately capturing peptide functional signals while minimizing errors.

### 2.5. Case Study

**Figure 6:**
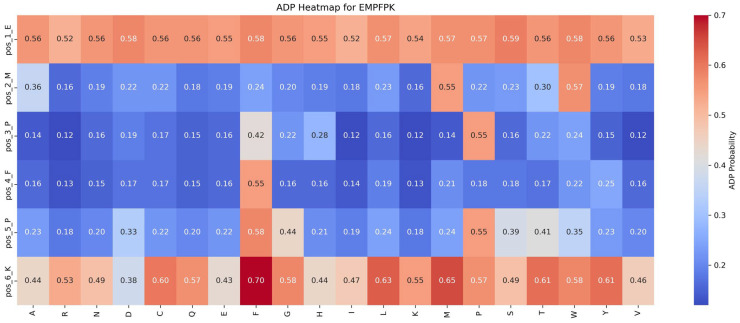
Impact of saturation mutagenesis on ADP function in the EMPFPK peptide from the ADP–AHP subset.

**Figure 7:**
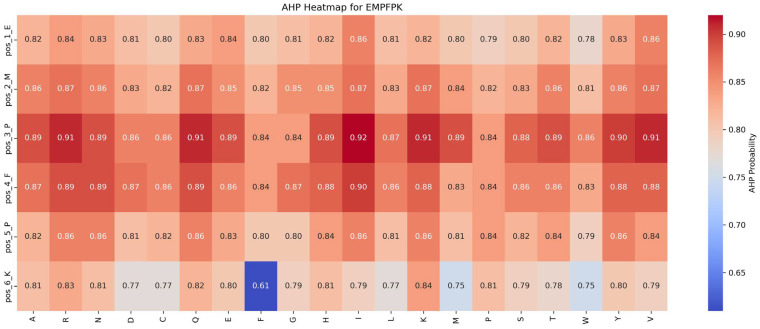
Impact of saturation mutagenesis on AHP function in the EMPFPK peptide from the ADP–AHP subset.

**Figure 8:**
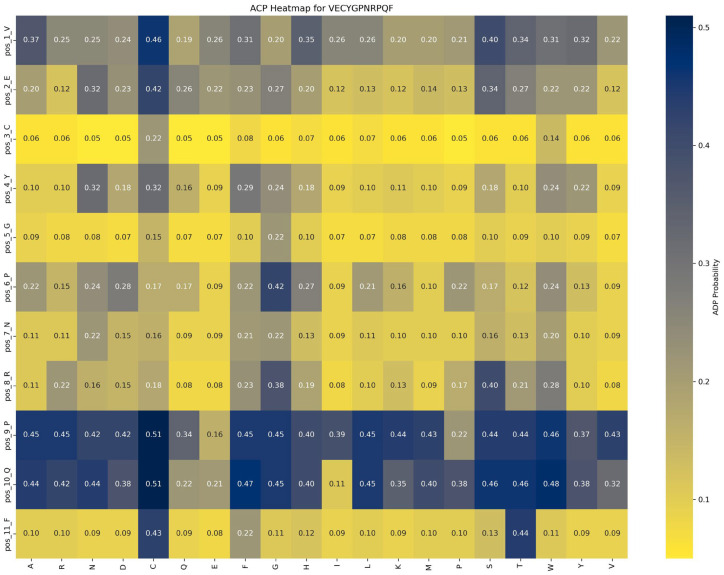
Impact of saturation mutagenesis on ADP function in the VECYGPNRPQF peptide from the ACP–AHP subset.

**Figure 9:**
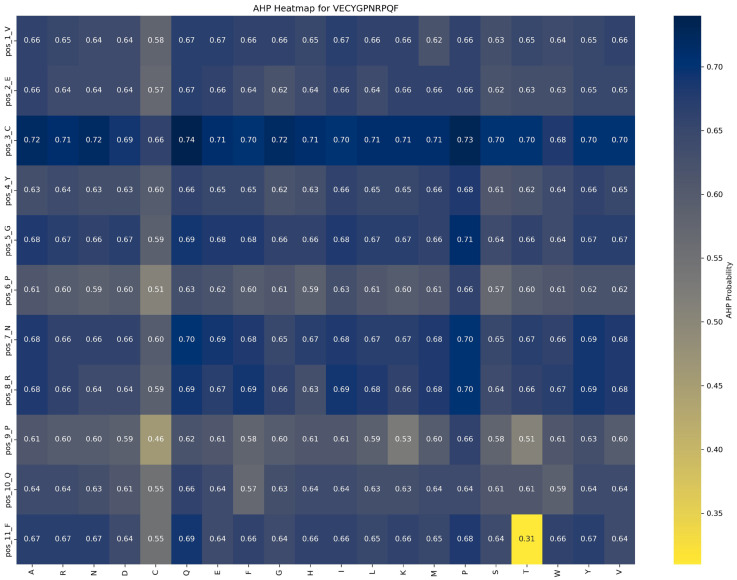
Impact of saturation mutagenesis on AHP function in the VECYGPNRPQF peptide from the ACP–AHP subset.

We performed saturation mutagenesis on multifunctional peptide tag data to investigate how mutations at various positions affect individual peptides and their combinations. The results of 86,970 mutations across four subsets (ACP–AHP: 210, ACP–AMP: 71,890, ADP–AHP: 2030, ADP–AIP: 12,840) are available at https://github.com/Zhou-Jianren/Multifunctional-peptide-classification/tree/main (accessed on 18 December 2024). The subsequent analysis focuses on saturation-induced mutations in two-peptide combinations, as illustrated in Figure 6, Figure 7, Figure 8 and Figure 9.

**(1)**  **Functional impact of saturation mutagenesis on ADP and AHP in the EMPFPK peptide**

This section assesses the impact of mutations on ADP and AHP functionalities by analyzing saturation mutagenesis data for the EMPFPK peptide at each position, as shown in Figure 6 and Figure 7.

Impact of mutations on ADP function: An analysis of Figure 6 reveals that the P → F substitution at position 5 significantly enhances ADP function, increasing the ADP function value from 0.55 to 0.58 (+0.03). This mutation likely improves the peptide’s binding affinity with the ADP receptor or stabilizes its structure, resulting in functional enhancement. Consequently, in the peptide design, the P → F substitution at position 5 should be prioritized to optimize ADP function while avoiding detrimental mutations at critical positions such as pos_2_M and pos_4_F to preserve functional stability.

Impact of mutations on AHP function: Figure 7 shows that the AHP function remains largely stable and resistant to mutations. For instance, the P → I mutation at position 3 results in a modest increase in AHP function, from 0.84 to 0.92 (+0.08). Most other mutations affect AHP function minimally, with changes within ±0.05, highlighting AHP’s robustness against mutations. While the P → F mutation at position 5 significantly enhances the ADP function, its impact on AHP function is limited, suggesting potential for the dual optimization of both functions.

Based on these findings, the following strategies are recommended for functional optimization: (1) Prioritize Beneficial Mutations: Implement mutations that enhance function or have minimal impact. For example, the P → F substitution at position 5 improves ADP function by 0.03 while minimally affecting AHP function (decrease of 0.04), making it an ideal candidate for optimization. (2) Avoid Detrimental Mutations: refrain from mutations that suppress function, such as M → R at position 2 and F → Y at position 4, which significantly reduce ADP function by 0.39 and 0.30, respectively.

By following these strategies, peptide design can be optimized to enhance desired functionalities while maintaining overall stability and effectiveness.

**(2)**  **Functional impact of saturation mutagenesis on ACP and AHP in the VECYGPNRPQF peptide**

Saturation mutagenesis analyses, depicted in Figure 8 and Figure 9, reveal that ACP function is more sensitive to mutations, particularly at position 1_V. In contrast, AHP function demonstrates higher stability and minimal sensitivity to mutations.

Impact of mutations on ACP function: An analysis of Figure 8 highlights the significant impact of the P → G substitution at position 6_P in the VECYGPNRPQF peptide on ACP function. Specifically, this mutation increases ACP function from 0.22 to 0.42, a 0.20 increase, underscoring the critical role of proline at this position. The P → G mutation likely enhances peptide binding affinity with the ACP receptor or stabilizes the peptide structure, leading to a marked functional improvement. Thus, in peptide design, prioritizing beneficial mutations like P → G at position 6_P is recommended to optimize ACP function while avoiding detrimental mutations at key positions such as 2_C and 5_G to maintain functional stability.

Impact of mutations on AHP function: Figure 9 demonstrates that AHP function in the VECYGPNRPQF peptide is more stable and less sensitive to mutations. For example, the P → E substitution at position 6_P causes a minor reduction in AHP function, from 0.66 to 0.62, a change of 0.04, indicating that mutations at this site have a limited effect on AHP activity. This suggests that AHP function exhibits high tolerance to mutations at position 6_P. Additionally, mutations at other positions result in changes within a ±0.05 range, further reinforcing the stability and resistance of AHP function to mutation.

Based on these findings, the following optimization strategies are proposed: (1) Prioritize beneficial mutations: Implement mutations that enhance function or have minimal impact. For example, the P → G substitution at position 6_P improves ACP function by 0.20 while minimally affecting AHP function (decrease of 0.04), making it an ideal candidate for optimization. (2) Avoid function-suppressing mutations: refrain from mutations that suppress function, such as E → R at position 2 and F → D at position 11, which significantly reduce ACP function by 0.10 and 0.13, respectively. (3) Dual optimization strategy: focus on mutations that enhance ACP function without adversely affecting AHP function, such as the P → G substitution at position 6_P, to achieve synergistic optimization of multifunctional peptides.

Additional saturation mutagenesis experiments on the LHLPLP and AKWVGDLTLCRWR peptides are presented in Figures S1–S4, with detailed analyses documented in Texts S2 and S3. The saturation mutagenesis strategy not only optimizes the functionality of multifunctional peptides but also ensures their stability, facilitating their synergistic optimization. This approach provides essential guidance for the precise design of multifunctional peptides, thereby advancing their application and development in the biomedical field.

## 3. Materials and Methods

### 3.1. Benchmark Datasets

This study compiled a comprehensive dataset of multifunctional bioactive peptides from Refs. [24,26,27]. The dataset comprises 6115 peptide sequences categorized into five functional groups: antimicrobial peptides (AMP, 2409), anti-inflammatory peptides (AIP, 1678), antihypertensive peptides (AHP, 868), anticancer peptides (ACP, 646), and antidiabetic peptides (ADP, 514). Additionally, 198 peptides exhibit multiple functions. Data distribution analysis indicates that most peptides possess a single biological function, with a minor subset demonstrating dual functionalities. Notably, no peptides were identified with more than two biological activities. Detailed distribution information is provided in Table S13.

**Figure 10:**
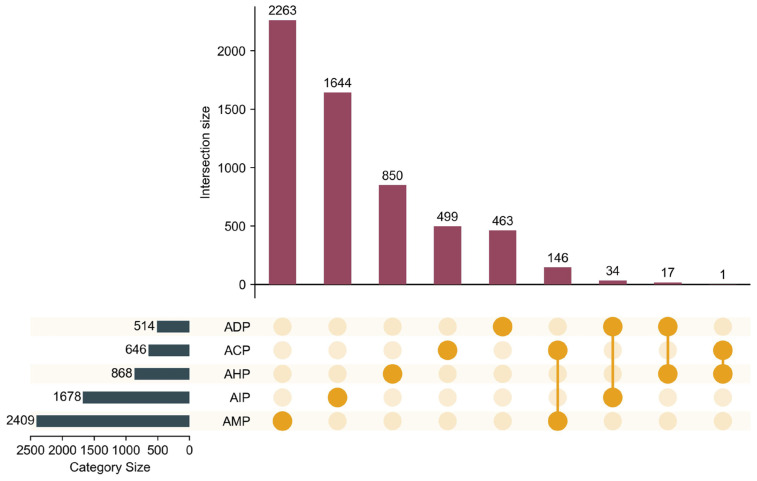
Distribution of bioactive peptides across functional categories. Note: Among these, yellow indicates the specific function of the peptide, categorized into single-function and dual-function types. Red denotes the number of peptides associated with each function, while dark blue signifies the number of peptides possessing a particular function.

To ensure data quality, a deduplication process was performed to eliminate redundant sequences. Post-deduplication, the number of single-functional peptides decreased as follows: ACP from 646 to 499, ADP from 514 to 463, AHP from 868 to 850, AIP from 1678 to 1644, and AMP from 2409 to 2263. The count of multifunctional peptides remained at 198. A summary of the refined dataset is presented in Table 2, with its distribution illustrated in Figure 10.

As shown in Table 2, the cleaned dataset includes 5719 single-functional peptides. AMP constitutes the largest category, representing 39.6% of the dataset, significantly surpassing other functional groups. In contrast, ADP accounts for the smallest portion at 8.1%. Multifunctional peptides make up 3.5% of the dataset, highlighting their relative rarity. Figure 1 visually depicts the distribution, emphasizing the predominance of AMP and AIP over ACP, AHP, and ADP. An intersection analysis in Figure 10 reveals that multifunctional peptides primarily exhibit dual biological activities, with certain activity pairs occurring more frequently.

For model development and evaluation, the cleaned dataset was randomly split into a training set (80%) and a testing set (20%). This partitioning ensures balanced data representation, enhances model generalizability, and facilitates robust performance evaluation in subsequent predictive analyses.

### 3.2. Feature Representation

ESM-2 [10] is an advanced protein language model built on the Transformer architecture, specifically engineered to extract essential biological insights from unlabeled protein sequences. Utilizing multi-head attention mechanisms and self-supervised learning, ESM-2 effectively captures long-range dependencies, outperforming traditional models such as RNNs and CNNs [28]. This proficiency makes it particularly suitable for tasks involving protein function prediction and structural analysis [29,30].

ProtT5 [19], adapted from the T5 framework, is specialized for protein sequence processing, emphasizing functional and structural predictions. By leveraging multi-head self-attention and a text-to-text modeling approach, ProtT5 interprets protein sequences as structured textual data, facilitating efficient feature extraction [31]. This methodology has significantly advanced protein analysis and understanding [32].

RoBERTa [9], an optimized variant of BERT, incorporates several enhancements, including larger training datasets, extended training durations, and an improved dynamic masking strategy, to achieve superior feature extraction capabilities. In this study, RoBERTa is employed through transfer learning and fine-tuning to identify and extract critical features from peptide sequences, thereby enhancing the accuracy and robustness of functional predictions [33,34].

Collectively, these models utilize state-of-the-art deep learning and self-supervised learning techniques to comprehensively extract meaningful features from protein sequences. Their ability to generate detailed and accurate representations provides a robust foundation for advancing the prediction of multifunctional peptides, effectively addressing the inherent complexity of peptide function analysis with precision and efficiency.

### 3.3. Model

#### 3.3.1. Workflow of the Proposed MFP-MFL Model

This study introduces MFP-MFL, a multi-feature, multi-label learning framework engineered to predict the functionalities of multifunctional bioactive peptides. The framework comprises four essential modules: data preprocessing, feature extraction, graph classification, and model integration, each enhancing the robustness and precision of the predictive system.

**Figure 11:**
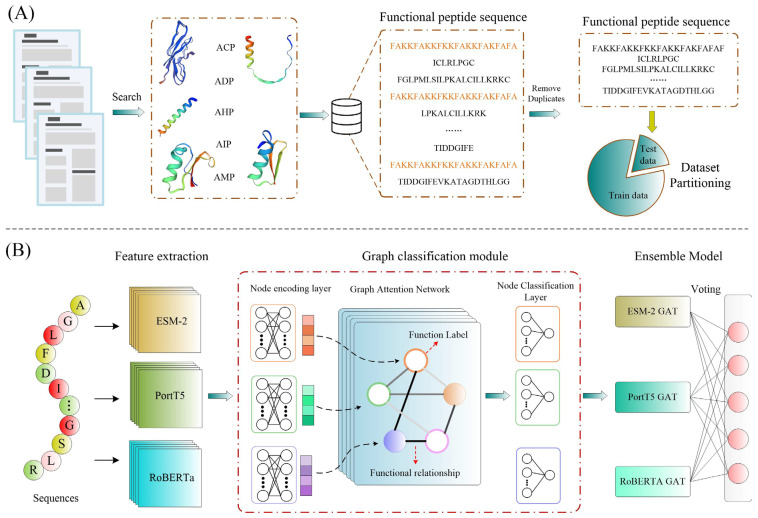
Comprehensive pipeline of the MFP-MFL framework. (**A**) Data preprocessing and dataset partitioning. (**B**) Workflow of the MFP-MFL model.

The data preprocessing module (Figure 11A) ensures the integrity of input data by eliminating duplicate peptide sequences and partitioning the dataset into training (80%) and testing (20%) subsets. This step is vital for maintaining data consistency and establishing a reliable basis for subsequent model training and evaluation.

In the feature extraction module (Figure 11B), three advanced transformer-based protein language models (including ESM-2 [10], ProtT5 [19], and RoBERTa [9]) are employed to derive deep sequence-level features. These models utilize self-attention mechanisms to process peptide sequences as structured text composed of 20 amino acid residues. By embedding complex sequence relationships with high fidelity, the extracted features encapsulate essential functional patterns, providing representations for downstream tasks.

In this study, the graph classification module employs a Graph Attention Network (GAT) to analyze and model the complex associations among peptide functional labels. Within this framework, peptide functional features are represented as nodes, while the relationships among these features are depicted as edges. These relationships reflect underlying biological correlations between different functional attributes. By utilizing a multi-head attention mechanism, GAT iteratively updates the information of each node and its neighboring nodes, thereby more accurately capturing the intricate interrelationships among peptide functions.

For instance, consider the sequence GFMKYIGPLIPHAVKAISDLI, whose functional labels are represented by a binary vector [1,1,0,0,0]. Each bit corresponds to a specific function: AMP, ACP, ADP, AHP, and AIP. A “1” indicates that the peptide possesses the given function, while a “0” signifies its absence. In our model, these functions are treated as nodes, and their potential biological associations are represented as the edges connecting them. For example, if certain functions frequently co-occur biologically, the model uses attention mechanisms to strengthen the weights of these associations. This approach enables the GAT to identify features in peptide sequences that are critical for predicting multifunctionality.

To further address the variability inherent in multifunctional peptide data, we incorporated adversarial training into the optimization process. This strategy enhances the model’s robustness and generalization, enabling more reliable performance across diverse datasets.

Finally, the model integration module aggregates predictions from multiple sub-models (e.g., ESM-2_GAT, ProtT5_GAT, and RoBERTa_GAT) using a weighted ensemble approach. This strategy not only capitalizes on the strengths of individual sub-models but also improves prediction stability and overall accuracy, ensuring consistent performance across various functional tasks.

#### 3.3.2. Graph Classification Module

The graph classification module is the core component of the model designed to transform multi-label prediction tasks into graph-based node classification problems. It comprises three essential submodules: a node encoder, a GAT network, and a node classifier. These submodules collaboratively establish a robust framework for functional prediction.

The node encoder maps peptide sequence features to node representations, which are subsequently processed by the GAT. This involves a series of linear transformations and dropout regularization to ensure the consistency and robustness of the node features, as defined below:

(1) $$h_{i}=dropout(W_{i}\cdot p)$$

where $h_{i}\in {\mathbb{R}}^{d\prime }$ is the encoded feature vector of node *i*, $W_{i}\in {\mathbb{R}}^{d\prime \times d}$ is a learnable weight matrix, and $p\in {\mathbb{R}}^{d}$ represents the raw feature vector of the peptide sequence. By standardizing feature dimensions to 128, the encoder ensures uniform node representations across the dataset, facilitating compatibility with subsequent graph-based computations.

The GAT submodule employs graph neural networks enhanced with an attention mechanism to model the intricate dependencies between functional labels [35,36]. By assigning learnable attention weights, the GAT identifies and emphasizes the most relevant relationships between nodes. The feature aggregation process for node *i* is described as follows:

(2) $$h_{i}^{\prime }=\sigma \left(\sum_{j\in N\left(i\right)}\alpha_{ij}\cdot Wh_{j}\right)$$

where $h_{i}^{\prime }$ denotes the updated feature of node *i*, σ is a nonlinear activation function, $N\left(i\right)$ represents the set of neighboring nodes of *i*, and $\alpha_{ij}$ quantifies the attention weight between nodes *i* and *j*. The attention coefficients are computed using the following equations:

(3) $$e_{ij}=LeakyReLU(a^{T}[Wh_{i}∥Wh_{j}])$$

(4) $$\alpha_{ij}=\frac{\exp(e_{ij})}{\sum_{k\in N(i)}\exp(e_{ik})}$$

where $e_{ij}$ represents the unnormalized attention score between nodes *i* and *j*, $a^{T}$ is a learnable vector used for combining features, and ∥ denotes the concatenation operation. The *LeakyReLU* activation function introduces non-linearity, enhancing the model’s ability to capture complex feature relationships. To further improve expressiveness, the GAT integrates multi-head attention mechanisms [37], aggregating multiple attention heads as follows:

(5) $$h_{i}^{\prime }={\|}_{k=1}^{K}\sigma \left(\sum_{j\in N(i)}\alpha_{ij}^{k}W^{k}h_{j}\right)$$

where *K* is the number of attention heads, and $\alpha_{ij}^{k}$ and $W^{k}$ are the attention coefficient and transformation matrix for the *k*-th attention head, respectively. This multi-head mechanism provides diverse perspectives on the relationships between nodes, improving the robustness of feature aggregation [38].

The node classifier generates the final functional predictions for each node by transforming the aggregated node features into label probabilities. The output is calculated as:

(6) $$result_{i}=sigmoid(W_{r}\cdot h_{i}^{\prime })$$

where $W_{r}$ is the weight matrix of the classification layer, and $h_{i}^{\prime }$ represents the final feature vector for node *i*. The sigmoid function ensures that the predicted values are normalized into probabilities, facilitating multi-label classification tasks.

#### 3.3.3. FGM Adversarial Training

To enhance model robustness and mitigate overfitting in sequence-based function prediction tasks, this study employs the Fast Gradient Method (FGM) [39] for adversarial training. FGM introduces adversarial perturbations to peptide sequence embeddings, exposing the model to more challenging input conditions during training and improving its generalization capability [40]. The implementation procedure is outlined below:

**Gradient computation**: For a given peptide sequence embedding *e* and its true functional label *y*, the gradient of the loss function with respect to the embedding is computed. This gradient *g* [41] quantifies the sensitivity of the loss to changes in the embedding under the current model parameters $\hat{\theta }$. The gradient *g* of the loss function $L\left(e,y\right)$ is formulated as:

(7) $$g=\nabla_{e}\log p(y|e;\hat{\theta })$$

Here, $p(y|e;\hat{\theta })$ denotes the conditional probability of *y* given the embedding *e* and the fixed model parameters $\hat{\theta }$.

**Generation of adversarial perturbations**: Utilizing the computed gradient, a small perturbation $r_{adv}$ is generated and added to the original embedding. The magnitude of the perturbation is controlled by the hyperparameter ε, ensuring it remains within a reasonable range [42]. The perturbation is defined as:

(8) $$r_{adv}=-\varepsilon \frac{g}{{\left\|g\right\|}_{2}}$$

where ε is a hyperparameter that scales the perturbation, and ${\left\|g\right\|}_{2}$ represents the L2 norm of gradient *g*.

**Generation of adversarial embeddings**: the adversarial perturbation $r_{adv}$ is added to the original embedding *e*, resulting in a new adversarial embedding $e_{adv}$:

(9) $$e_{adv}=e+r_{adv}$$

**Adversarial training**: The model is trained using both the original embedding *e* and the adversarial embedding $e_{adv}$, minimizing the initial loss $L_{init}$ and the adversarial loss $L_{adv}$ simultaneously. This approach enhances the model’s robustness and generalization capacity [43]. The functions are defined as follows:

(10) $$L_{init}(\theta )=-\frac{1}{N}\sum_{i=1}^{N}y_{i}\log p(y_{i}|e_{i};\theta )$$

(11) $$L_{adv}(\theta )=-\frac{1}{N}\sum_{i=1}^{N}y_{i}\log p(y_{i}|e_{i}+r_{adv,i};\theta )$$

By incorporating adversarial training, the model achieves improved resilience to variations in input embeddings, enabling it to maintain stability and predictive accuracy even under challenging conditions. This strategy effectively enhances the model’s ability to generalize across diverse input scenarios.

## 4. Conclusions

Multifunctional bioactive peptide prediction remains a challenging and complex issue in bioinformatics. In response, we introduced MFP-MFL, an innovative framework designed to address the shortcomings of existing methods in predicting peptide multifunctionality. By integrating three state-of-the-art protein language models (including ESM-2, ProtT5, and RoBERTa), MFP-MFL extracts robust features and employs a weighted ensemble approach to enhance predictive accuracy. At its core, the model leverages a GAT, reframing the multi-label prediction task as a graph node classification problem. This design effectively captures the intricate relationships among functional labels, leading to improved performance metrics. The experimental evaluations reveal that MFP-MFL achieves superior results across key indicators, including accuracy (0.799), coverage (0.821), and precision (0.786), with an Absolute error of just 0.086. Furthermore, we benchmarked MFP-MFL against other established methods for multifunctional peptide prediction, spanning both machine learning (e.g., CLR, RBRL) and deep learning (e.g., MLBP, RoBERTa) techniques. In the machine learning comparison, MFP-MFL demonstrated substantial improvements, achieving an accuracy of 0.786 versus RBRL’s 0.649. Similarly, in the deep learning comparison, MFP-MFL exceeded the performance of RoBERTa, which achieved an accuracy of 0.769. These results underline the framework’s predictive advantages. In addition to its superior benchmark performance, MFP-MFL was further validated through a case study involving 86,970 mutations. This investigation confirmed the model’s exceptional ability to predict functional changes resulting from peptide sequence mutations. Overall, MFP-MFL sets a new standard for predicting multifunctional bioactive peptides, offering a powerful and reliable tool for this challenging domain.

## Figures and Tables

**Table 1:** Performance comparison of GAT using individual features and ensemble feature sets.

Features	Precision	Coverage	Accuracy	Absolute True	Absolute False
ESM-2	0.746	0.780	0.738	0.687	0.104
PortT5	0.770	0.815	0.759	0.690	0.098
RoBERTa	0.786	0.800	0.772	0.731	0.093
Ensemble	**0.799**	**0.821**	**0.786**	**0.737**	**0.086**

Note: ESM-2, PortT5, and RoBERTa represent single-feature ensemble models. Ensemble denotes the multi-feature ensemble model. The evaluation metrics presented in the table are computed using the formulas outlined in the multi-label classification module of Text S1, and the specific calculations can be found in Text S5. Unless explicitly stated otherwise, all metrics reported in the table are derived following the multi-label classification framework. The corresponding confusion matrix for this table can be found in Table S4. Among them, the ensemble model is the MFP-MFL model.

**Table 2:** The cleaned bioactive peptide datasets.

Function Type	Functional Category	Number of Peptides
Single function	ACP	499
	ADP	463
	AHP	850
	AIP	1644
	AMP	2263
	total	5719
Multifunction	Two functions	198

Note: The redundant multifunctional peptides from both the training and test sets in the original dataset have been selected and are presented in Tables S14 and S15.

## Data Availability

The benchmark datasets and Python source code used in this study are available on https://github.com/Zhou-Jianren/Multifunctional-peptide-classification (accessed on 18 December 2024).

## Supplementary Materials

The following supporting information can be downloaded at: https://www.mdpi.com/article/10.3390/ijms26031317/s1.
