# Supplementary material for: MFP-MFL: Leveraging Graph Attention and Multi-Feature Integration for Superior Multifunctional Bioactive Peptide Prediction
Source: Int J Mol Sci. 2025 Feb 4;26(3):1317. doi: 10.3390/ijms26031317 (PMC11818429; doi:10.3390/ijms26031317)
Supplement: Supplementary file 1 [file ijms-26-01317-s001.zip › ijms-3404496-supplementary.pdf]

Supplementary Materials

MFP-MFL: Leveraging Graph Attention and Multi-Feature Integration for Superior Multifunctional Bioactive Peptide Prediction

Fang Ge [1], Jianren Zhou [2], Ming Zhang [2] and Dong-Jun Yu [3,*]

[1] State Key Laboratory of Flexible Electronics (LoFE), Institute of Advanced Materials (IAM), Nanjing University of Posts and Telecommunications, 9 Wenyuan Road, Nanjing 210023, China

[2] School of Computer, Jiangsu University of Science and Technology, 666 Changhui Road, Zhenjiang 212100, China

[3] School of Computer Science and Engineering, Nanjing University of Science and Technology, 200 Xiaolingwei, Nanjing 210094, China

[*] Correspondence: njyudj@njust.edu.cn

Supplementary Texts

Supplementary Text S1. Evaluation metrics

(1) Evaluation Metrics for Multi-Label Classification

To rigorously evaluate the performance of the proposed model and facilitate meaningful comparisons with other methods, this study employs five widely recognized metrics commonly used in multi-functional classification tasks: **precision, coverage**, **accuracy**, **absolute true**, and **absolute false**. These metrics are mathematically defined as follows:

$$Precision = \frac{1}{S}\sum_{n=1}^{S}\frac{\|C_n \cap C_n^*\|}{\|C_n^*\|} \tag{S1}$$

$$Coverage = \frac{1}{S}\sum_{n=1}^{S}\frac{\|C_n \cap C_n^*\|}{\|C_n\|} \tag{S2}$$

$$Accuracy = \frac{1}{S}\sum_{n=1}^{S}\frac{\|C_n \cap C_n^*\|}{\|C_n \cup C_n^*\|} \tag{S3}$$

$$Absolute\ true = \frac{1}{S}\sum_{n=1}^{S}\Delta(C_n, C_n^*) \tag{S4}$$

$$\Delta(C_n, C_n^*) = \begin{cases} 1, & if\ C_n^*\ is\ identical\ to\ C_n \\ 0, & other \end{cases} \tag{S5}$$

$$Absolute\ false = \frac{1}{S}\sum_{n=1}^{S}\frac{\|C_n \cup C_n^*\| - \|C_n \cap C_n^*\|}{T} \tag{S6}$$

where S represents the total number of peptide samples, C_n denotes the true label set for the *n-th* sample, C_n^* represents the predicted label set for the *n-th* sample, and T is the total number of distinct labels across all samples.

(2) Evaluation Metrics for Binary Classification

To provide a more granular evaluation of the model's performance for individual peptide functionalities, this study further employs additional metrics, including sensitivity (SEN), specificity (SPE), accuracy (ACC), and the Matthews Correlation Coefficient (MCC). The definitions of these metrics are as follows:

$$SEN = \frac{TP}{TP+FN} \tag{S7}$$

$$SPE = \frac{TN}{TN+FP} \tag{S8}$$

$$ACC = \frac{TP+TN}{TP+FP+TN+FN} \tag{S9}$$

$$MCC = \frac{TP \times TN - FP \times FN}{\sqrt{(TP+FP)(TP+FN)(TN+FP)(TN+FN)}} \tag{S10}$$

where, *TP*, *TN*, *FP*, and *FN* refer to true positives, true negatives, false positives, and false negatives, respectively. By integrating these evaluation metrics, this study provides a comprehensive framework for assessing the model's effectiveness across multiple functional peptide prediction tasks. These metrics not only ensure the reliability and robustness of the evaluation but also enable objective comparisons with other predictive models[1–3]. This rigorous evaluation approach contributes to a deeper understanding of the model's performance, strengths, and potential areas for improvement.

Supplementary Text S2. Impact of Mutations on ADP and AHP Functions Illustrated in Figures S1-S2

(1) Impact of Mutations on ADP Function

The analysis of data presented in **Figure S1** highlights the significant impact of the H→R substitution at position pos_2_H in the LHLPLP peptide on ADP function. Specifically, this mutation reduces ADP function from 0.58 to 0.22, a decrease of 0.36, underscoring the critical role of histidine at this position. The H→R substitution likely interferes with key peptide-receptor interactions, leading to a marked decrease in function. Therefore, peptide design should prioritize avoiding such detrimental mutations at pos_2_H to maintain ADP function stability.

(2) Impact of Mutations on AHP Function

Figure S2 demonstrates that the L→R substitution at position pos_1_L results in a slight increase in AHP function, with the value rising from 0.79 to 0.83 (an increase of 0.04). However, this mutation also strongly inhibits ADP function, reducing its value from 0.58 to 0.16, a drop of 0.42. While this substitution slightly improves AHP function, it simultaneously suppresses ADP function, highlighting a trade-off between optimizing one function while compromising the other.

(3) Functional Optimization Strategies

Based on the findings, the following strategies for functional optimization are recommended: avoid function-suppressing mutations, for instance, the H→R substitution at pos_2_H significantly diminishes ADP function (a decrease of 0.39) and should be avoided.

By employing these optimization strategies, it is possible to enhance ADP function while preserving AHP function stability, ultimately achieving synergistic optimization of multifunctional peptides. This approach provides essential guidance for the precise design of multifunctional peptides, facilitating their potential applications in the biomedical field.

Supplementary Text S3. Impact of Mutations on ACP and AMP Functions Illustrated in Figures S3-S4

Saturation mutagenesis analysis presented in Figures S3 and S4 reveals that ACP function is more sensitive to mutations, particularly at positions pos_12_W and pos_11_R, whereas AMP function demonstrates greater stability with minimal mutation impact.

(1) Impact of Mutations on ACP Function

Data in **Figure S3** show that the K→W substitution at position pos_2_K in the AKWVGDLTLCRWR peptide has a substantial effect on ACP function. Specifically, the ACP function decreases from 0.51 to 0.24, a reduction of 0.27. This highlights the critical role of lysine at position pos_2_K in maintaining ACP function. The K→W mutation likely disrupts the peptide`s binding capacity to the ACP receptor, leading to a marked decline in function. Consequently, such mutations at pos_2_K should be avoided during peptide design to preserve ACP function stability.

(2) Impact of Mutations on AMP Function

As shown in **Figure S4**, the K→W substitution at pos_2_K has a minimal effect on AMP function, with the AMP value increasing slightly from 0.68 to 0.73 (a change of 0.05). This indicates that the mutation at pos_2_K has a limited impact on AMP function, demonstrating AMP's high tolerance to mutations at this position. Furthermore, mutations at most other positions affect AMP function by no more than ±0.05, further confirming AMP's stability and resistance to mutation-induced alterations.

(3) Functional Optimization Strategies

Based on the above findings, the following strategies are recommended: (1) Select mutations that enhance function or have minimal impact. For example, the A→R substitution at pos_1_A results in a small increase in AMP function (from 0.68 to 0.71) and a negligible effect on ACP function (from 0.51 to 0.52), making it a promising candidate for optimization. (2) Avoid mutations that suppress function: The K→W substitution at pos_2_K significantly reduces ACP function (by 0.27) and should be avoided. (3) Adopt a dual optimization strategy: Prioritize mutations that have minimal impact on AMP function but contribute to optimizing ACP function, such as the A→R substitution at pos_1_A, to achieve synergistic optimization of multifunctional peptides.

By implementing these strategies, ACP function can be enhanced while AMP function remains stable, leading to the synergistic optimization of multifunctional peptides. This approach provides critical insights for the precise design of multifunctional peptides, supporting their applications in biomedical research and development.

Supplementary Text S4. Comparison model description

CLR [4] employs a calibrated label ranking approach, addressing the issue of uncalibrated utility scales inherent in traditional methods. By introducing manually calibrated labels, it effectively distinguishes between relevant and irrelevant labels, and extends pairwise comparison learning to multi-label scenarios. The approach integrates the strengths of pairwise preference learning and correlation-based classification, significantly enhancing the expressiveness of label ranking.

RAKEL [5] is an ensemble method for multi-label classification. This approach constructs an ensemble model by randomly selecting a small subset of labels and learning single-label classifiers for each element of the power set of these subsets. By doing so, RAKEL captures label correlations at the subtask level, while ensuring manageable label numbers and a sufficient amount of training data.

MLDF [6] applies the deep forest framework to multi-label learning problems. It introduces two key mechanisms: metric-aware feature reuse and metric-aware layer growth. The former enables the model to reuse high-quality representations from previous layers, while the latter guides the progressive increase in model complexity based on performance metrics. This dual approach not only mitigates overfitting but also optimizes multi-label evaluation metrics as specified by the user.

RBRL [7] is a multi-label classification model that integrates Ranking Support Vector Machines (Rank-SVM) with Binary Relevance (BR) and incorporates robust low-rank learning. This approach combines the advantages of Rank-SVM in minimizing ranking loss, while addressing the challenges of BR, such as ignoring label correlations and handling class imbalance. Additionally, RBRL retains BR's ability to perform single-step learning and minimize Hamming loss, overcoming the error accumulation issues caused by the stacked threshold handling of Rank-SVM. Furthermore, under the assumption of a low-dimensional label space, RBRL utilizes low-rank constraints to capture higher-order label correlations, with kernelization support to enable nonlinear multi-label classification. Optimization efficiency is enhanced through the use of two accelerated proximal gradient methods.

MLBP [3] is a multi-label deep learning approach designed to simultaneously predict various functions of multifunctional bioactive peptides, including anticancer, antidiabetic, antihypertensive, anti-inflammatory, and antimicrobial properties. Unlike traditional methods that rely on biological and physicochemical features, MLBP uses peptide sequence vectors as input. It replaces these conventional features by learning dense, continuous feature vectors through an embedding layer, and further extracts and optimizes features by combining convolutional neural network layers with bidirectional gated recurrent unit layers.

MPMABP [8] is a deep learning method based on Convolutional Neural Networks (CNN) and Bidirectional Long Short-Term Memory (Bi-LSTM), designed to identify multiple bioactivities of bioactive peptides. The MPMABP model stacks five CNNs of varying sizes and employs residual networks to ensure that information is preserved throughout the network.

RoBERTa [1] is a pre-trained method for bioactive peptide recognition that significantly enhances model performance in identifying various functional peptides, such as anticancer, antidiabetic, antihypertensive, anti-inflammatory, and antimicrobial peptides. This improvement is achieved through extensive pre-training on large-scale protein sequences, which allows the model to better capture the underlying patterns and relationships within peptide data.

Supplementary Text S5. Evaluation matrix Calculation Methodology

The evaluation of evaluation matrix follows two distinct methodologies, depending on whether the task is multi-label classification (for Section 2.1-2.4, except for Figure 5) or binary classification (for Figure 5 and Tables S11-12). The multi-label classification approach employs Formulas (S1)–(S6) from Text S1, whereas the binary classification task relies on Formulas (S7)–(S9).

For multi-label classification tasks, evaluation values are computed based on set-based evaluation metrics, ensuring alignment with standard methodologies in biologically active peptide classification studies. This approach is consistent with existing literature [9,10] to maintain comparability across studies. In contrast, for binary classification tasks, the evaluation follows a confusion matrix-based approach.

To avoid misinterpretation, evaluation matrix calculations for multi-label classification strictly adhere to Formulas (S1)–(S6), whereas binary classification evaluations use Formulas (S7)–(S9). Based on the reviewer's suggestion, step-by-step example matrix calculations are provided below.

S1. Evaluation Metrics Calculation for Multi-Label Classification Tasks

The following outlines the multi-label classification calculation process: Using the MFP-MFL model (Ensemble feature model) as an example (referencing the “Ensemble” row in Table 1), we first obtain all predicted peptide labels generated by the model and compare them with the corresponding ground truth labels. The evaluation is based on the union and intersection of the predicted and actual labels.

For instance:

Predicted labels: [1,0,1,0,0]

True labels: [1,1,0,0,0]

The union of the sets contains three elements, while the intersection contains one element. The accuracy for this specific sample is then computed as:

$$\frac{|C_n \cap C_n^*|}{|C_n \cup C_n^*|} = \frac{1}{3}$$

If the intersection is empty (i.e., no matching labels), the accuracy for that sample is assigned as zero. The same computation is applied to all 1184 peptides, and the final metric values are obtained by averaging across all 1185 samples.

The predicted and ground truth labels for the MFP-MFL model (Ensemble feature model) can be accessed at: https://github.com/Zhou-Jianren/Multifunctional-peptide-classification/tree/main/labelValue (accessed on 18 December 2024), where 0 represents "False" and 1 represents "True" in the data file.

(1) Calculation of Precision:

$$Precision = \frac{1}{S}\sum_{n=1}^{S}\frac{\left\| C_n \cap C_n^* \right\|}{\left\| C_n^* \right\|}$$

$$\text{where,}\ \sum_{n=1}^{S}\frac{\left\| C_n \cap C_n^* \right\|}{\left\| C_n^* \right\|} = 946.5$$

$$Precision = \frac{946.5}{1185} \approx 0.799$$

(2) Calculation of Coverage:

$$Coverage = \frac{1}{S}\sum_{n=1}^{S}\frac{\left\| C_n \cap C_n^* \right\|}{\left\| C_n \right\|}$$

$$\text{where,}\ \sum_{n=1}^{S}\frac{\left\| C_n \cap C_n^* \right\|}{\left\| C_n \right\|} = 973$$

$$Coverage = \frac{973}{1185} \approx 0.821$$

(3) Calculation of Accuracy:

$$Accuracy = \frac{1}{S}\sum_{n=1}^{S}\frac{\left\| C_n \cap C_n^* \right\|}{\left\| C_n \cup C_n^* \right\|}$$

$$\text{where,}\ \sum_{n=1}^{S}\frac{\left\| C_n \cap C_n^* \right\|}{\left\| C_n \cup C_n^* \right\|} = 930.83$$

$$Accuracy = \frac{930.83}{1185} \approx 0.786$$

(4) Calculation of Absolute True:

$$Absolute\ true = \frac{1}{S}\sum_{n=1}^{S}\Delta(C_n, C_n^*),\quad \Delta(C_n, C_n^*) = \begin{cases} 1, & if\ C_n^*\ is\ identical\ to\ C_n \\ 0, & other \end{cases}$$

$$\text{where}\ \sum_{n=1}^{S}\Delta(C_n, C_n^*) = 873$$

$$Absolute\ true = \frac{873}{1185} \approx 0.737$$

(5) Calculation of Absolute False:

$$Absolute\ false = \frac{1}{S}\sum_{n=1}^{S}\frac{\left\| C_n \cup C_n^* \right\| - \left\| C_n \cap C_n^* \right\|}{T}$$

where $\frac{\left\| C_n \cup C_n^* \right\| - \left\| C_n \cap C_n^* \right\|}{T} = 102.20$

$$Absolute\ false = \frac{102.20}{1185} \approx 0.086$$

S2. Evaluation Metrics Calculation for Binary Classification Tasks (For Figure 5 and Tables S11-S12)

In the binary classification task analysis presented in Figure 5 (taking ACP of MFP-MFL model as an example, as depicted in Figure 5A), we compute several evaluation metrics, including ACC, SEN, SPE, and MCC. For binary classification, we employ Formulas (S7)–(S10) from Supplementary Text S1 to calculate these metrics.

Example Calculation (ACP, Figure 5, Table S12, Ensemble Feature Model): For ACP, based on Figure 5A, the provided confusion matrix values are: TP = 77, TN = 1013, FP = 41, FN = 54

(1) Calculation of ACC:

$$ACC = \frac{TP + TN}{TP + FP + TN + FN} = \frac{77 + 1013}{77 + 1013 + 41 + 54} \approx 0.920$$

(2) Calculation of SEN:

$$SEN = \frac{TP}{TP + FN} = \frac{77}{77 + 54} \approx 0.588$$

(3) Calculation of SPE:

$$SPE = \frac{TN}{TN + FP} = \frac{1013}{1013 + 41} \approx 0.961$$

(4) Calculation of MCC:

$$\begin{aligned} MCC &= \frac{(TP \times TN) - (FP \times FN)}{\sqrt{(TP + FP)(TP + FN)(TN + FP)(TN + FN)}} \\ &= \frac{(77 \times 1013) - (41 \times 54)}{\sqrt{(77 + 41)(77 + 54)(1013 + 41)(1013 + 54)}} \\ &= \frac{75787}{\sqrt{118 \times 131 \times 1054 \times 167}} \approx 0.630 \end{aligned}$$

Supplementary Figures

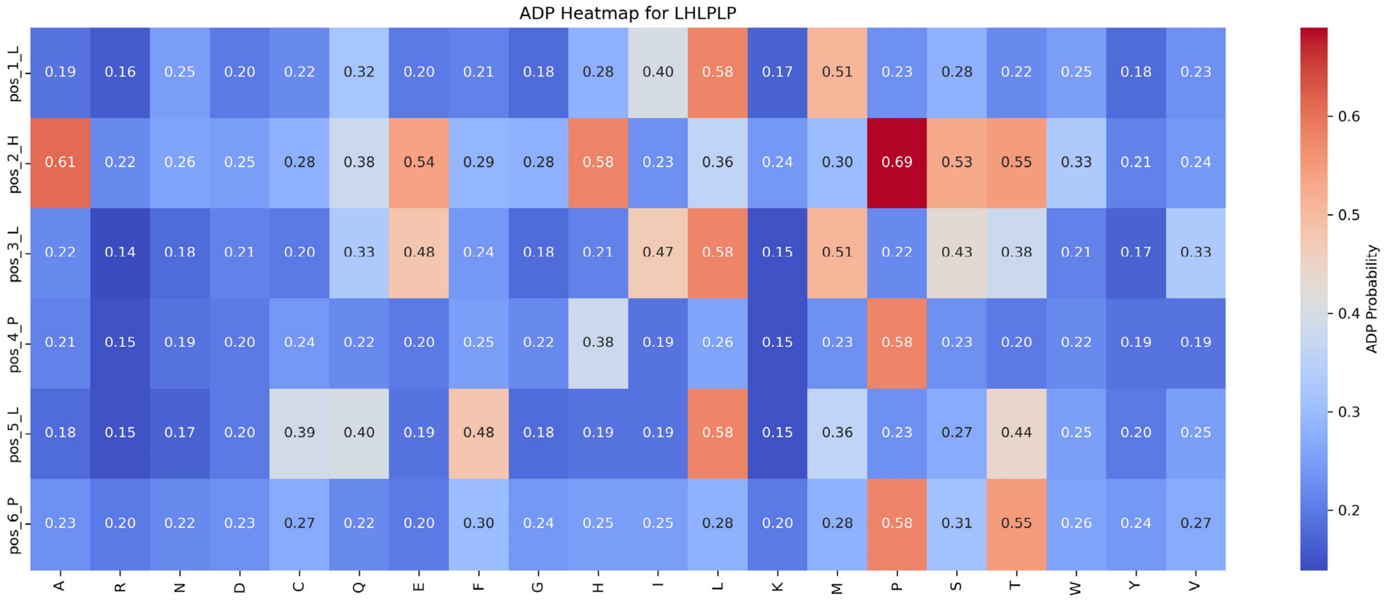

Figure S1. Impact of saturation mutagenesis on ADP function in the LHLPLP peptide from the ADP-AHP subset.

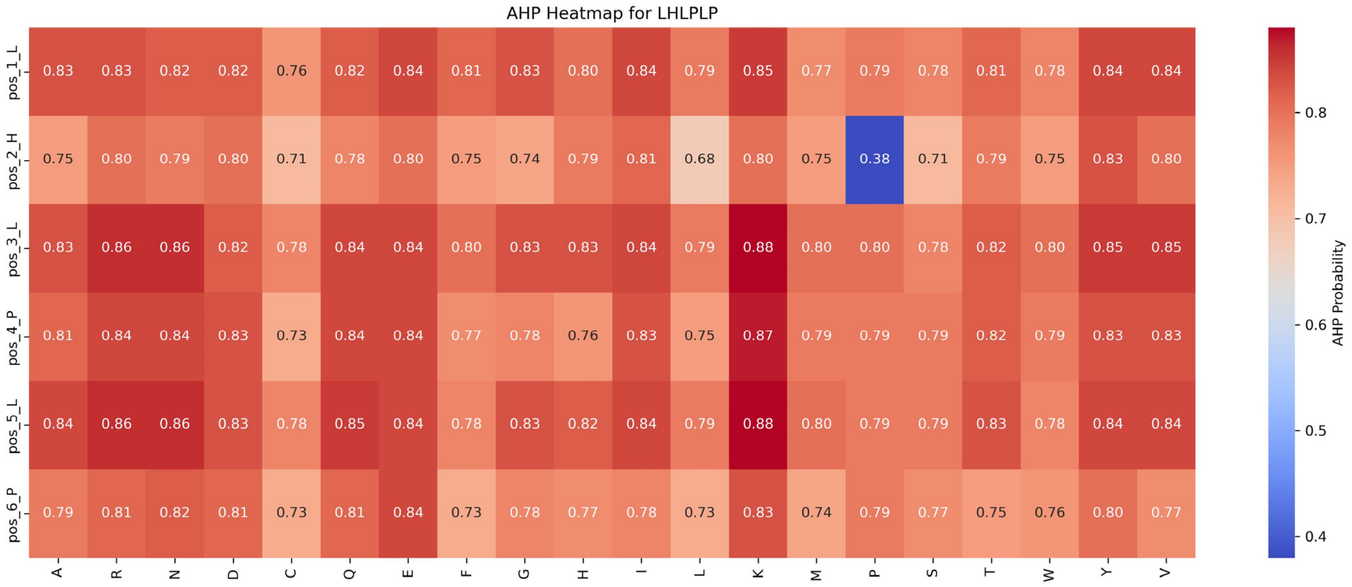

Figure S2. Impact of saturation mutagenesis on AHP function in the LHLPLP peptide from the ADP-AHP subset.

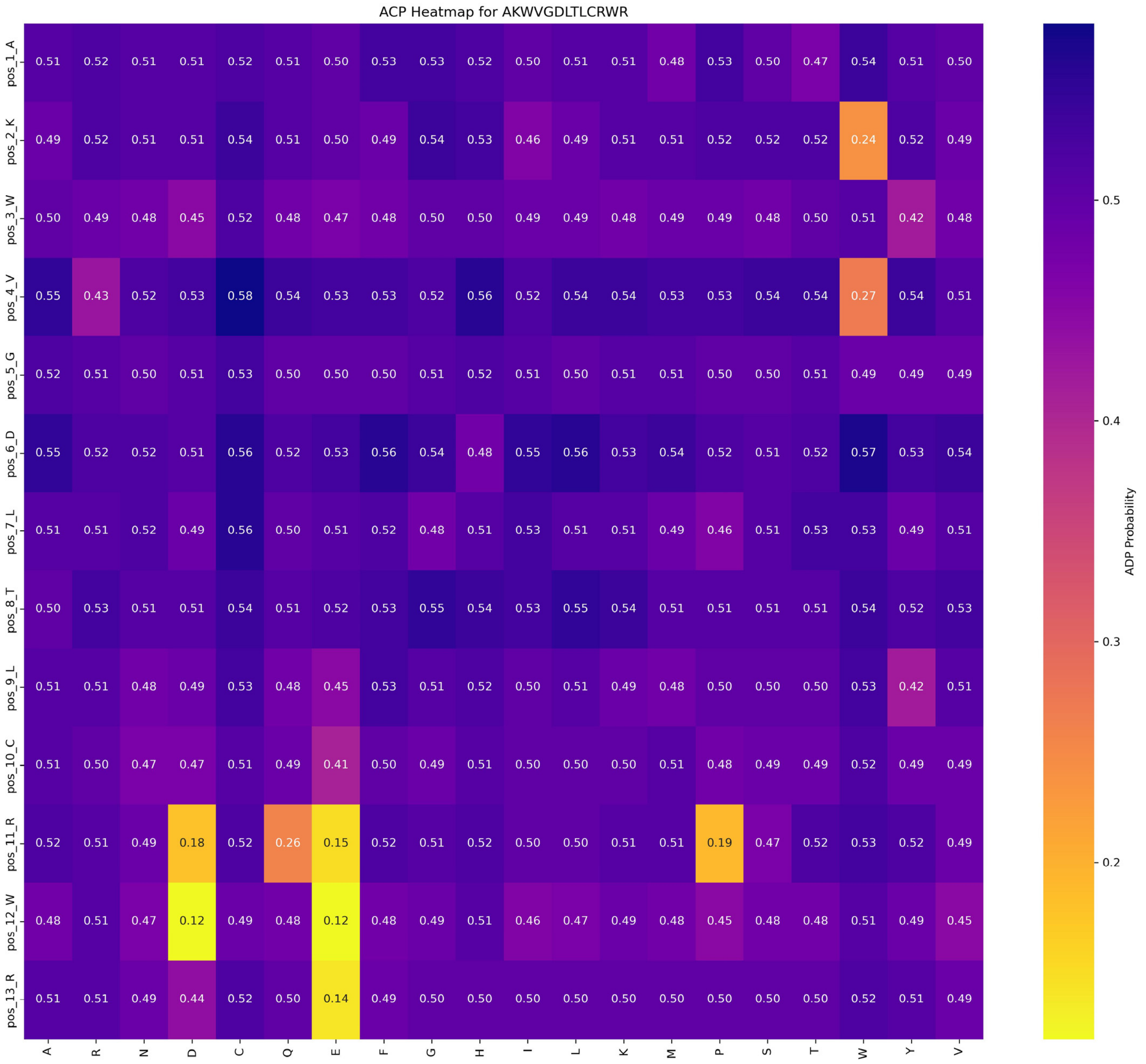

Figure S3. Impact of saturation mutagenesis on ACP function in the AKWVGDLTLCRWR peptide from the ACP-AMP subset.

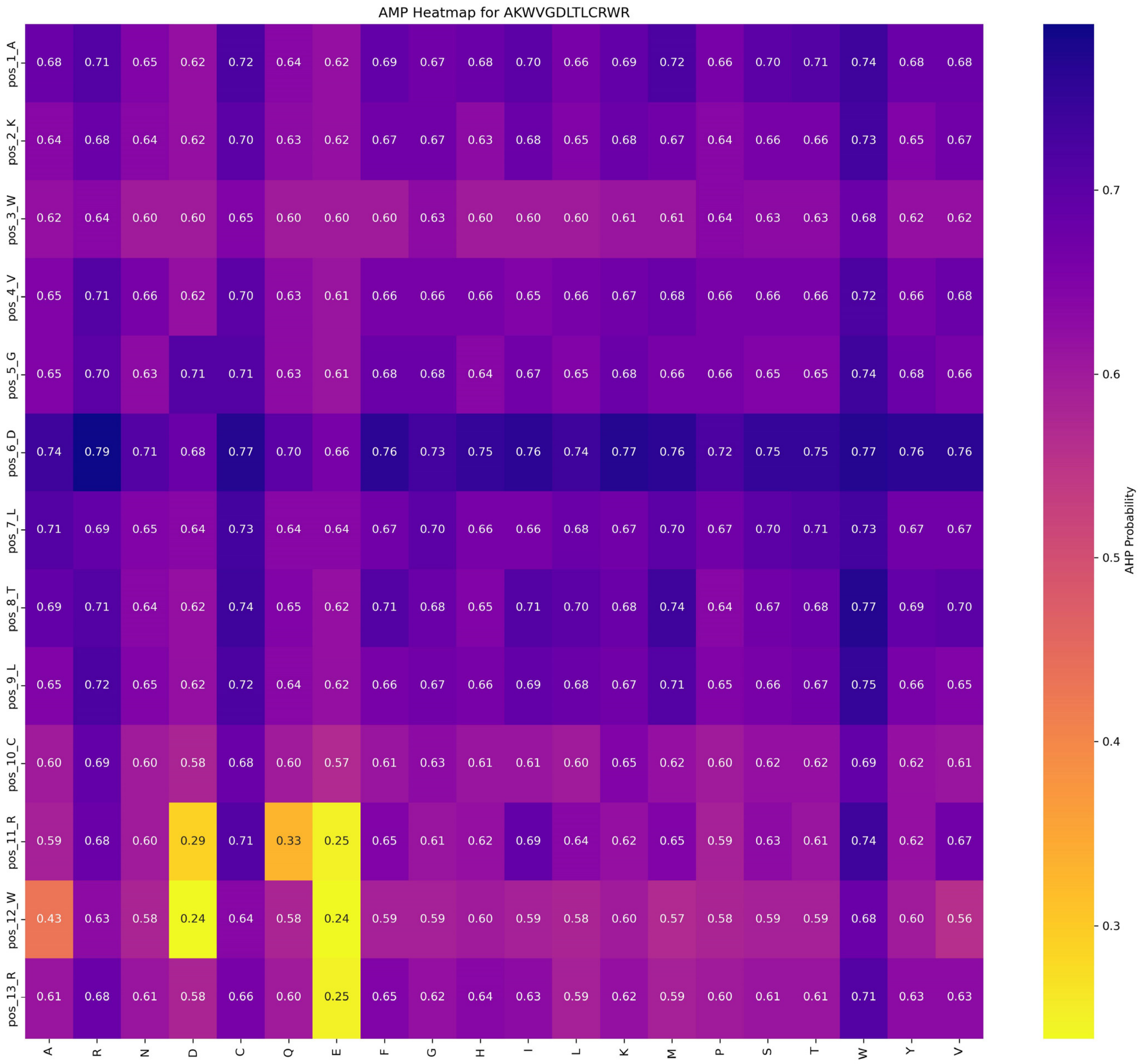

Figure S4. Impact of saturation mutagenesis on AMP function in the AKWVGDLTLCRWR peptide from the ACP-AMP subset.

Supplementary Tables

Table S1. Performance of different features on the GAT model.

Features	Precision	Coverage	Accuracy	Absolute true	Absolute false
ESM-2	0.740±0.010	0.784±0.018	0.731±0.010	0.669±0.023	0.107±0.003
PortT5	0.764±0.004	0.798±0.016	0.752±0.003	0.693±0.018	0.100±0.002
RoBERTa	**0.784±0.003**	**0.800±0.009**	**0.770±0.003**	**0.727±0.004**	**0.093±0.001**

Note: The evaluation metrics listed in the table are calculated using the formulas provided in the multi-label classification module of Text S1. Unless otherwise specified, all metrics presented in the tables are derived using the multi-label classification methodology. The bold text in the table represents the optimal value for each indicator.

Table S2. Experimental results of KNN model on different features

Features	Precision	Coverage	Accuracy	Absolute true	Absolute false
ESM-2	0.690	0.691	0.678	0.651	0.114
PortT5	0.711	0.706	0.697	0.676	0.108
RoBERTa	**0.791**	**0.796**	**0.777**	**0.743**	**0.090**

Note: The evaluation metrics listed in the table are calculated using the formulas provided in the multi-label classification module of Text S1. Unless otherwise specified, all metrics presented in the tables are derived using the multi-label classification methodology. The confusion matrices for the five labels of each feature model can be found in **Table S3**. The bold text in the table represents the optimal value for each indicator.

Table S3. Confusion matrix of KNN model on different features.

Feature	Peptide type	TN	FP	FN	TP
ESM-2	AMP	638	63	137	347
	ACP	1014	40	57	74
	ADP	1055	28	76	26
	AHP	978	33	37	137
	AIP	730	121	83	251
PortT5	AMP	632	69	127	357
	ACP	1015	39	60	71
	ADP	1053	30	62	40
	AHP	983	28	27	147
	AIP	750	101	94	240
RoBERTa	AMP	638	63	91	393
	ACP	1011	43	57	74
	ADP	1038	45	59	43
	AHP	979	32	20	154
	AIP	761	90	35	299

Table S4. Confusion matrix of GAT using individual features and ensemble feature sets.

Feature	Peptide type	TN	FP	FN	TP
ESM-2	AMP	618	83	90	394
	ACP	1015	39	61	70
	ADP	1077	6	87	15
	AHP	968	43	16	158
	AIP	704	147	16	318
PortT5	AMP	625	76	85	399
	ACP	1022	32	63	68
	ADP	1058	25	61	41
	AHP	971	40	15	159
	AIP	732	119	35	299
RoBERTa	AMP	634	67	88	396
	ACP	1009	45	55	76
	ADP	1037	46	56	46
	AHP	985	26	23	151
	AIP	760	91	37	297
Ensemble	AMP	633	68	77	407
	ACP	1013	41	54	77
	ADP	1040	43	57	45
	AHP	981	30	16	158
	AIP	752	99	26	308

Note: we calculated the model performance using different features, as shown in Table 1, by employing the formulas provided in Text S1. Table S4 presents the confusion matrices for the five labels of each feature model. Among them, the Ensemble model is the MFP-MFL model.

Table S5. Performance of different features and Ensemble method on the MLP model.

Features	Precision	Coverage	Accuracy	Absolute true	Absolute false
ESM-2	0.770±0.004	0.796±0.008	0.757±0.003	0.704±0.012	0.098±0.002
PortT5	0.754±0.008	0.781±0.010	0.741±0.008	0.687±0.016	0.104±0.004
RoBERTa	0.788±0.004	0.799±0.006	0.774±0.004	0.736±0.004	0.091±0.001
Ensemble	**0.797**	**0.818**	**0.784**	**0.736**	**0.087**

Note: ESM-2, PorT5, and RoBERTa correspond to the outcomes of single-feature experiments, while Ensemble refers to the multi-feature ensemble model. The bold text in the table represents the optimal value for each indicator.

Table S6. Performance of different features and Ensemble method on the GCN model.

Features	Precision	Coverage	Accuracy	Absolute true	Absolute false
ESM-2	0.737±0.008	0.774±0.011	0.728±0.007	0.674±0.017	0.108±0.004
PortT5	0.761±0.006	0.812±0.017	0.750±0.005	0.678±0.018	0.101±0.004
RoBERTa	0.787±0.003	0.805±0.005	0.773±0.002	0.728±0.007	0.093±0.001
fusion	**0.795**	**0.807**	**0.782**	**0.744**	**0.087**

Note: ESM-2, PorT5, and RoBERTa correspond to the outcomes of single-feature experiments, while Ensemble refers to the multi-feature ensemble model. The bold text in the table represents the optimal value for each indicator.

Table S7. Performance of model fusion on MLP.

Round/Fusion	Precision	Coverage	Accuracy	Absolute Ture	Absolute False
0	0.790	0.801	0.775	0.734	0.091
1	0.790	0.801	0.777	0.739	0.090
2	0.792	0.800	0.779	0.744	0.089
3	0.791	0.804	0.777	0.736	0.091
4	0.789	0.801	0.776	0.738	0.090
5	0.788	0.798	0.774	0.737	0.091
6	0.788	0.799	0.774	0.735	0.091
7	0.788	0.798	0.774	0.736	0.091
8	0.789	0.797	0.776	0.741	0.091
9	0.792	0.801	0.777	0.739	0.090
fusion	0.791	0.804	0.777	0.737	0.091

Note: This dataset is composed of ESM-2, PortT5 and RoBERTa feature fusion to reduce the dimension and form new features

Table S8. Performance of model fusion on GAT.

id	Precision	Coverage	Accuracy	Absolute ture	Absolute false
0	0.790	0.804	0.777	0.736	0.091
1	0.787	0.808	0.773	0.724	0.093
2	0.784	0.792	0.770	0.733	0.091
3	0.791	0.795	0.777	0.746	0.090
4	0.789	0.797	0.774	0.737	0.091
5	0.791	0.801	0.777	0.738	0.091
6	0.775	0.783	0.761	0.726	0.096
7	0.783	0.800	0.770	0.728	0.092
8	0.779	0.789	0.765	0.727	0.096
9	0.790	0.800	0.776	0.739	0.091
fusion	0.789	0.796	0.774	0.738	0.091

Note: This dataset is composed of ESM-2, PortT5 and RoBERTa feature fusion to reduce the dimension and form new features

Table S9. Performance of model fusion on GCN.

id	Precision	Coverage	Accuracy	Absolute ture	Absolute false
0	0.788	0.804	0.774	0.730	0.092
1	0.791	0.806	0.777	0.735	0.092
2	0.792	0.800	0.777	0.741	0.090
3	0.793	0.801	0.778	0.741	0.088
4	0.788	0.813	0.775	0.724	0.093
5	0.789	0.804	0.775	0.732	0.092
6	0.788	0.806	0.774	0.729	0.093
7	0.786	0.798	0.772	0.732	0.092
8	0.789	0.799	0.775	0.738	0.092
9	0.789	0.806	0.776	0.732	0.092
fusion	0.792	0.800	0.777	0.739	0.090

Note: This dataset is composed of ESM-2, PortT5 and RoBERTa feature fusion to reduce the dimension and form new features

Table S10. Performance comparison of our proposed method with the state-of-the-art methods on the MFBP dataset.

Method	Precision	Coverage	Accuracy	Absolute true	Absolute false
CLR	0.667	0.677	0.666	0.655	0.133
RAKEL	0.649	0.648	0.648	0.647	0.141
MLDF	0.649	0.649	0.648	0.646	0.119
RBRL	0.650	0.651	0.649	0.646	0.140
MLBP	0.709	0.717	0.708	0.699	0.106
MPMABP	0.728	0.749	0.727	0.704	0.101
RoBERTa	0.781	0.786	0.769	**0.741**	0.092
MFP-MFL	**0.799**	**0.821**	**0.786**	0.737	**0.086**

Note: The bold text in the table represents the optimal value for each indicator.

Table S11. Performance of single-function peptides on the RoBERTa model.

Peptide	AUC	AUPR	F1	ACC	SEN	SPE	MCC	Random Accuracy
ACP	0.775	0.455	0.636	0.927	0.580	0.970	0.599	0.818
ADP	0.648	0.252	0.416	0.924	0.314	0.982	0.404	0.878
AHP	0.933	0.727	0.843	0.950	0.908	0.958	0.816	0.733
AIP	0.865	0.674	0.792	0.875	0.841	0.888	0.705	0.580
AMP	0.853	0.758	0.827	0.860	0.818	0.889	0.709	0.518

Note: The data in this table are computed using the binary classification methodology outlined in Text S1. The confusion matrix for the model can be accessed in Table S16, with the random accuracy calculated as $[(TN + FP)(TN + FN) + (TP + FP)(TP + FN)] / N^2$. Obviously, the ACC values substantially exceed Random Accuracy across Table S11. For example, in AHP classification task, the RoBERTa model achieves ACC=0.950, which is 21,7% percentage points higher than the corresponding Random Accuracy.

Table S12. Performance of single-function peptides on the MFP-MFL model.

Peptide	AUC	AUPR	F1	ACC	SEN	SPE	MCC	Random Accuracy
ACP	0.930	0.699	0.619	0.920	0.588	0.961	0.575	0.812
ADP	0.899	0.538	0.474	0.916	0.441	0.960	0.430	0.852
AHP	0.987	0.926	0.873	0.961	0.908	0.970	0.851	0.741
AIP	0.949	0.840	0.831	0.895	0.922	0.884	0.764	0.568
AMP	0.943	0.928	0.849	0.878	0.841	0.903	0.746	0.518

Note: The data in this table are computed using the binary classification methodology outlined in Text S1. The formula of Random Accuracy is $[(TN + FP)(TN + FN) + (TP + FP)(TP + FN)] / N^2$. Similar to Table S11, the ACC values in Table S12 consistently surpass the Random Accuracy, exemplified by the AMP classification task where ACC=0.878 exceeds the Random Accuracy of 0.518 by 36.0 percentage points.

Table S13. Distribution of the Original Dataset.

No.	Functional category	Number of peptides
1	ACP	646
2	ADP	514
3	AHP	868
4	AIP	1678
5	AMP	2409
Total number of single functional category peptides		6115
Total number of multi-functional category peptides		198

Table S14. Information on duplicate sequences removed from the original train subset.

AMP	ACP	ADP	AHP	AIP	Sequence
1	1	0	0	0	KVKVKVKVPPTKVKVKVK
1	1	0	0	0	FIHHIIGGLFSAGKAIHRLIRRRRR
1	1	0	0	0	ATAVDFGPHGLLPIRPIRIRPLCGKDKS
1	1	0	0	0	PNEVNRLAHLRLH
1	1	0	0	0	GLFDIVKKIAGHIAGSI
0	0	1	0	1	DRGEKPASPAVQPDAALQRLAAVL
0	0	1	0	1	ECDFQEFMAFVAMVTTACHEFFEHE
1	1	0	0	0	FMPILSCSRFKRC
1	1	0	0	0	YCAYYSPRHKTTF
1	1	0	0	0	SPLGYGFAVRNSG
1	1	0	0	0	AGCIKNGGRCNASAGPPYCCSSYCFQIAGQSYGVCKNR
1	1	0	0	0	FLPPSPWKETFRTS
1	1	0	0	0	FNRGGYNFGKSVRHVVDAIGSVAGILKSIR
0	0	1	0	1	LIAFTSEHSHFSLKKGAAAL
0	0	1	1	0	VRGPFP
1	1	0	0	0	NYQWVPYQGRVPYPRGGLLKLLKKLLKKLLKL
0	0	1	0	1	KAMVALIDVFHQYSGREGDK
1	1	0	0	0	ELCEKASQTWSGTCGKTKHCDDQCKSWEGAAHGACHVRDGKHMCFCYFNC
1	1	0	0	0	ATYYGNGLYCNKEKCWVDWNQAKGEIGKIIVNGWVNHGPWAPRR
0	0	1	0	1	LAKLSDGVAVLKVGG
1	1	0	0	0	FKVQNQHGQVVKIFHH
0	0	1	1	0	LNVPGE
1	1	0	0	0	GLLQTIKEKLESLESLAKGIVSGIQA
1	1	0	0	0	CAAGTACTCAGTGTGGA
0	0	1	1	0	FTESQS
1	1	0	0	0	CCTAAGCCCTTGTGGTGTGT
1	1	0	0	0	KSCCPSTTARNIYNTCRLTGASRSVCASLSGCKIISGSTCDSGWNH
1	1	0	0	0	FFPNVASVPGQVLLKKIFCAISKKC
1	1	0	0	0	GFKLKGMARISCLPNGQWSNFPPKCIRECAMVSS
1	1	0	0	0	FLLFPLMCKIQGKC
1	1	0	0	0	MRKEFHNVLSSGQLLADKRPARDYNRK
1	1	0	0	0	ATRVVYCNRRSGSVVGGDDTVYYEG
1	1	0	0	0	EFTNVSCTTSKECWSVCQRLHNTSRGKCMNKKCRCYS
0	0	1	0	1	KDYRAYYTFLNFMSNVGDPR
0	0	1	1	0	VLVLDTDYK
0	0	1	0	1	SEHIWCEDFLVRSFYLKNVQ
0	0	1	1	0	APFPE
1	1	0	0	0	ATCKAECPTWDSVCINKKPCVACCKKAKFSDGHCSKILRRCLCTKEC
0	0	1	0	1	NPVEIRRGVMLAVDAVIAEL
0	0	1	0	1	VPRLPEQGSSSRAEDSPEG
1	1	0	0	0	ESVFSKIGNAVGPAAYWILKGLGNMSDVNQADRINRKKH
0	0	1	1	0	IPIQY
1	1	0	0	0	DYDWSLRGPPKCATYGQKCRTWSPRNCCWNLRCKAFRCRPR
1	1	0	0	0	CSTNTFSLSDYWGNKGNWCTATHECMSWCK
1	1	0	0	0	FLPLLAGLAANFLPTIICKISYKC
1	1	0	0	0	GFGALFKFLAKKVAKTVAKQAAKQGAKYVVNKQME
0	0	1	0	1	KPTQAFVKQHLCGPHL
1	1	0	0	0	FMPIIGRLMSGSL
1	1	0	0	0	AVPDVAFNAYG
1	1	0	0	0	ILGPVISTIGGVLGGLLKNL
1	1	0	0	0	EADEPLWLYKGDNIERAPTTADHPILPSIIDDVKLDPNRRYA
1	1	0	0	0	EIRLPEPFRFPSPTVPKPIDIDPILPHPWSPRQTYPIIARRS
1	1	0	0	0	FLPILINLIHKGLL

0	0	1	0	1	DGVTVAKSIDLKDKYKNIGA
0	0	1	1	0	FAQTQS
0	0	1	0	1	SRLSKVAPVIKARMMEYGTT
1	1	0	0	0	FLPAIAGILSQLF
1	1	0	0	0	LLGMIPLAISAISALSKL
1	1	0	0	0	DDTPSSRCGSGGWGPCLPIVDLLCIVHVTVGCSGGFGCCRIG
1	1	0	0	0	AFTCHCRRSCYSTEYSYGTCTVMGINHRFCCL
1	1	0	0	0	CTGAAGGTGCTGTCCCAGAT
1	1	0	0	0	FITLLLRKFICSITKKC
1	1	0	0	0	GETFDKLKEKLKTFYQKLVEKAEDLKGDLKAKLS
0	0	1	0	1	AALGIGTDSVILIKCDERGK
1	1	0	0	0	FLSLIPSLVGGSISAFK
1	1	0	0	0	AREASKSLIGTASCTCRRAWICRWGERHSGKCIDQKGSTYRLCCRR
1	1	0	0	0	GIGGVLLSAGKAALKGLAKVLAEKYAN
1	1	0	0	0	FAEPLPSEEEGESYSKEPPEMEKRYGGFM
1	1	0	0	0	AIKLVQSPNGNFAASFVLDGTKWIFKSKYYDSSKGYWVGIYEVWDRK
0	0	1	1	0	AAATP
0	0	1	0	1	KWCANPDWIHIDTTPFAGLV
1	1	0	0	0	CYTQYRKCQELTA
1	1	0	0	0	LKLKSIVSWAKKVL
1	1	0	0	0	GIPCAESCVWIPPCTITALMGCSCKNNVCYNN
0	0	1	0	1	EYVTLKKMREIIGWPGGSGD
0	0	1	0	1	DFWQMVWESGCTVIVMLTPLVEDGV
1	1	0	0	0	GCASRCKAKCAGRRCKGWASASFRGRCYCKCFRC
1	1	0	0	0	FLGGILNTITGLL
1	1	0	0	0	FLPLLLAGLPLKLCFLFKKC
1	1	0	0	0	GIGKFLHSAKKFGKAFVGEIMNS
1	1	0	0	0	YKQCHKKGGHCFPKEKICLPPSSDFGKMDCRWRWKCCKKGSG
1	1	0	0	0	FLPLILRKIVTAL
1	1	0	0	0	AKCIKNGKGCREDQGPPFCCSGFCYRQVGWARGYCKNR
1	1	0	0	0	ALWKTMLKKLGTMALHAGKAALGAAADTISQGTQ
1	1	0	0	0	CLGIGSCNDFAGCGYAVVCFW
0	0	1	0	1	SFYLKNVQTQETRTLTQFHF
0	1	0	1	0	VECYGPNRPQF
0	0	1	1	0	YPVEPF
1	1	0	0	0	HGVSGHGQHGVHG
0	0	1	0	1	DVMNILLQYVVKSFDRSTKV
1	1	0	0	0	FLPIASLLGKYL
1	1	0	0	0	FVPYNPPRPYQSKPFPSFPGHGPFNPKIQWPYPLPNPGH
1	1	0	0	0	DIQIPGIKKPTHRDIIIPNWNPNVRTQPWQRFGGNKS
1	1	0	0	0	GFLDIIKDTGKEFAVKILNNLKCKLAGGCPP
0	0	1	0	1	WYVMVTAALSYTISRMEESSVTL
1	1	0	0	0	GSLCGDTCFVLGCNDSSCSCNYPICVKD
1	1	0	0	0	ATRSYGNGVYCNNSKCWVNWGEAKENIAGIVISGWASGLAGMGH
1	1	0	0	0	DHYICAKKGGTCNFSPCPLFNRIEGTCYSGKAKCCIR
1	1	0	0	0	GLMDTIKGVAKTVAASWLDKLKCKITGC
0	0	1	1	0	LPYPY
1	1	0	0	0	AKKVFKRLEKLFSKIQNDK
1	1	0	0	0	ARLKKCFNKVTGYCRKKCKVGERYEIGCLSGKLCCAN
1	1	0	0	0	AIGSILGALAKGLPTLISWIKNR
1	1	0	0	0	FLSHIAGFLSNLF
1	1	0	0	0	GFFKKAWRKVKHAGRRVLDTAKGVGRHYVNNWLNRYR
1	1	0	0	0	FWGHIWNAVKRVGANALHGAVTGALS
1	1	0	0	0	FFPIGVFCKIFKTC
1	1	0	0	0	GEYCGESCYLIPCFTPGCYCVSRQCVNKN
1	1	0	0	0	GLFVGLAKVAAHNNPAIAEHFQA

1	1	0	0	0	FIGPIISALASLFG
1	1	0	0	0	SQETFSDLWKLLPEN
1	1	0	0	0	FWGALAKGALKLIPSLFSSFSKKD
1	1	0	0	0	GLVTSLIKGAGKLLGGLFGSVTGGQS
1	1	0	0	0	ALWKNMLKGIGKLAGKAALGAVKKLVGAES
1	1	0	0	0	GIGTKILGGVKTALKGALKELASTYAN
1	1	0	0	0	TCGTCCTGAGGAGAGAGAGC
1	1	0	0	0	AAKPMGITCDLLSLWKVGHAACAAHCLVLGDVGGYCTKEGLCVCKE
1	1	0	0	0	ENFFKEIERAGQRIRDAIISAAPAVETLAQAQKIIKGGD
1	1	0	0	0	GADFQECMKEHSQKQHQHQG
1	1	0	0	0	AANFGPSVFTPEVHETWQKFLNVVVAALGKQYH
0	0	1	0	1	HTPGVHMASLSVYLKTNVFL
0	0	1	1	0	EMPFPK
1	1	0	0	0	AKWVGDLTLCRWR
1	1	0	0	0	ACDTATCVTHRLAGLLSRSGGVVKNNFVPTNVGSKAF
0	0	1	0	1	LPGPSPAQLFQDSGLLYLAQE
0	0	1	1	0	VAGTWY
1	1	0	0	0	FLFPLITSFLSKVL
0	0	1	0	1	KKGAAALGIGTDSVI
1	1	0	0	0	DILTFEHYWAQLTS
1	1	0	0	0	GKWMSLLKHILK
1	1	0	0	0	FLGALIKGAIHGGRFIHGMIQNHH
0	0	1	0	1	YVVKSFDRSTKVIDFHYPNE
0	0	1	0	1	SKDQFEFALTAVAEEVNAILKA
1	1	0	0	0	DAEFRHDSGYEVHHQKLVFFAEDVGSNKGAIIGLMVGGVVIA
0	0	1	0	1	LYHVYEVNLVSEHIWCEDFL
1	1	0	0	0	YAIDTILLEIKNINAD
1	1	0	0	0	GAARKSIRLHRLYTWKATIYTR
1	1	0	0	0	FGLPMLSILPKALCILLKRKC
0	0	1	0	1	FLPLLALLALWEPKPTQA
1	1	0	0	0	FRGLAKLLKIGLKSFARVLKKVLPKAAKAGKALAKSMADENAIRQQNQ
1	1	0	0	0	GCSRWIIGIHGQICRD
1	1	0	0	0	ADTLACRQSHQSCSFVACRAPSVDIGTCRGGKLKCCKWAPSS
1	1	0	0	0	GFGCNGPWDEDDMQCHNHCKSIKGYKGGYCAKGGFVCKCY
0	0	1	1	0	LHLPLP
1	1	0	0	0	KSCCRNTWARNCYNVCRLPGTISREICAKKCDCKIISGTTCPSDYPK
1	1	0	0	0	GFFALIPKIISSPLFKTLLSAVGSALSSSGGQE
1	1	0	0	0	FKSWSFCTPGCAKTGSFNSYCC
1	1	0	0	0	VYINKLTPPCGTMYYACEAV
1	1	0	0	0	TESYFVFSVGM
1	1	0	0	0	KAYARIGNSYFK
1	1	0	0	0	GSSSGRGDSPA
0	0	1	0	1	KLKVESSPSRSDYINASPIIEHDP
1	1	0	0	0	FAVGLRAIKRALKKLRRGVRKVAKDL
1	1	0	0	0	FIITGLVRGLTKLF
0	0	1	0	1	GEALSTLVLNRLKVG
1	1	0	0	0	GFGSLLGKALRLGANVL
1	1	0	0	0	DVKGMKKAIKGILDCVIEKGYDKLAAKLKKVIQQLWE
0	0	1	0	1	SPSLWEIEFAKQLASV

Table S15. Information on duplicate sequences removed from the original test subset.

AMP	ACP	ADP	AHP	AIP	Sequence
0	0	1	0	1	REGDKHKLKKSELKEL
1	1	0	0	0	CTFTLPGGGGVCTLTSECIC
1	1	0	0	0	GRRKRKWLRRIGKGVKIIGGAALDHL
1	1	0	0	0	ELPKLPDDKVLIRSRSNCPKGKVWNGFDCKSPFAFS
1	1	0	0	0	EVWRLAEFLAMPP
1	1	0	0	0	ACYCRIGACVSGERLTGACGLNGRIYRLCCR
1	1	0	0	0	DQYKCLQHGGFCLRSSCPSNTKLQGTCKPDKPNCCKS
1	1	0	0	0	FFSASCVPGADKGQFPNLCRLCAGTGENKCA
1	1	0	0	0	RRRRRRRRGEDIIRNIARHLAQVGDSMDR
1	1	0	0	0	CIPMAWAVSWPHP
1	1	0	0	0	FLPAIVGAAAKFLPKIFCAISKKC
1	1	0	0	0	GRFKRFRKKFKKLFKKLSPVIPLLHLG
0	0	1	0	1	KFGADARALMLQGVDLLADA
1	1	0	0	0	ALKAALLAILKIVRVIKK
0	0	1	0	1	TGHMILAYMEDHLRNRDR
0	0	1	1	0	TPEVDDEALEK
1	1	0	0	0	LTAEHYAAQATS
1	1	0	0	0	LMMLILAMNRKDKKKEKK
1	1	0	0	0	FAKKFAKKFKKFAKKFAKFAFAF
0	0	1	0	1	KHKLKKSELKELINNELSHFLE
1	1	0	0	0	ADRGWIKTLTKDCPNVISSICAGTIITACKNCA
1	1	0	0	0	FFGSVLKLIPKIL
1	1	0	0	0	FLPVLAGIAAKVVPALFCKITKKC
1	1	0	0	0	DPVTYIRNGGICQYRCIGLRHKIGTCGSPFKCCK
1	1	0	0	0	FLSFPTTKTYFPHFDLSHGSAQVKGHGAK
1	1	0	0	0	FLPLIGRVLSGIL
1	1	0	0	0	GLFDKLKSLVSDF
1	1	0	0	0	GFMKYIGPLIPHAVKAISDLI
1	1	0	0	0	DTHFPICIFCCGCCHRSKCGMCCKT
1	1	0	0	0	KQLIRFLKRLDRNGGGKLLLKLLKKLLKLLKKK
0	0	1	1	0	LPVPQ
1	1	0	0	0	GTGLPMSERRKIMLMMR
0	0	1	1	0	LTDVEN
1	1	0	0	0	RRRRRRRRGNLWAAQRYGRELRRMSDEFVDSFKK
1	1	0	0	0	GFLDTFKNLALNAAKSAGVSVLNSLSCKLFKTC
1	1	0	0	0	EPHPDEFVGLM
1	1	0	0	0	CIKNGNGCQPNGSQNGCCSGYCHKQPGWVAGYCRRK
1	1	0	0	0	ATPATPTVAQFVIQGSTICLVC
0	0	1	1	0	GPFPILV
0	0	1	0	1	YEMVFDGKPQHTNVCFWYIP

Table S16. Confusion matrix of RoBERTa model.

Peptide type	TN	FP	FN	TP
AMP	623	78	88	396
ACP	1022	32	55	76
ADP	1063	20	70	32
AHP	968	43	16	158
AIP	756	95	53	281

References

1. Jiang, L.; Sun, N.; Zhang, Y.; Yu, X.; Liu, X. Bioactive Peptide Recognition Based on NLP Pre-Train Algorithm. *IEEE/ACM Trans. Comput. Biol. and Bioinf.* **2023**, *20*, 3809–3819, doi:10.1109/TCBB.2023.3323295.
2. Luo, J.; Zhao, K.; Chen, J.; Yang, C.; Qu, F.; Yan, K.; Zhang, Y.; Liu, B. Discovery of Novel Multi-Functional Peptides by Using Protein Language Models and Graph-Based Deep Learning 2023, 2023.04.14.536982.
3. Tang, W.; Dai, R.; Yan, W.; Zhang, W.; Bin, Y.; Xia, E.; Xia, J. Identifying Multi-Functional Bioactive Peptide Functions Using Multi-Label Deep Learning. *Briefings in Bioinformatics* **2022**, *23*, bbab414, doi:10.1093/bib/bbab414.
4. Fürnkranz, J.; Hüllermeier, E.; Loza Mencía, E.; Brinker, K. Multilabel Classification via Calibrated Label Ranking. *Machine learning* **2008**, *73*, 133–153.
5. Tsoumakas, G.; Vlahavas, I. Random K-Labelsets: An Ensemble Method for Multilabel Classification. In Proceedings of the European conference on machine learning; Springer, 2007; pp. 406–417.
6. Yang, L.; Wu, X.-Z.; Jiang, Y.; Zhou, Z.-H. Multi-Label Learning with Deep Forest. In *ECAI 2020*; IOS Press, 2020; pp. 1634–1641.
7. Wu, G.; Zheng, R.; Tian, Y.; Liu, D. Joint Ranking SVM and Binary Relevance with Robust Low-Rank Learning for Multi-Label Classification. *Neural Networks* **2020**, *122*, 24–39.
8. Li, Y.; Li, X.; Liu, Y.; Yao, Y.; Huang, G. MPMABP: A CNN and Bi-LSTM-Based Method for Predicting Multi-Activities of Bioactive Peptides. *Pharmaceuticals* **2022**, *15*, 707.
9. Tang, W.; Dai, R.; Yan, W.; Zhang, W.; Bin, Y.; Xia, E.; Xia, J. Identifying Multi-Functional Bioactive Peptide Functions Using Multi-Label Deep Learning. *Briefings in Bioinformatics* **2022**, *23*, bbab414, doi:10.1093/bib/bbab414.
10. Jiang, L.; Sun, N.; Zhang, Y.; Yu, X.; Liu, X. Bioactive Peptide Recognition Based on NLP Pre-Train Algorithm. *IEEE/ACM Trans. Comput. Biol. and Bioinf.* **2023**, *20*, 3809–3819, doi:10.1109/TCBB.2023.3323295.
